# The Clinical Application of MicroRNAs in Infectious Disease

**DOI:** 10.3389/fimmu.2017.01182

**Published:** 2017-09-25

**Authors:** Ruth E. Drury, Daniel O’Connor, Andrew J. Pollard

**Affiliations:** ^1^Oxford Vaccine Group, Centre for Clinical Vaccinology and Tropical Medicine, Department of Paediatrics, University of Oxford, The Churchill Hospital, Oxford, United Kingdom

**Keywords:** microRNAs, infection, epigenetics, biomarkers, vaccines

## Abstract

MicroRNAs (miRNAs) are short single-stranded non-coding RNA sequences that posttranscriptionally regulate up to 60% of protein encoding genes. Evidence is emerging that miRNAs are key mediators of the host response to infection, predominantly by regulating proteins involved in innate and adaptive immune pathways. miRNAs can govern the cellular tropism of some viruses, are implicated in the resistance of some individuals to infections like HIV, and are associated with impaired vaccine response in older people. Not surprisingly, pathogens have evolved ways to undermine the effects of miRNAs on immunity. Recognition of this has led to new experimental treatments, RG-101 and Miravirsen—hepatitis C treatments which target host miRNA. miRNAs are being investigated as novel infection biomarkers, and they are being used to design attenuated vaccines, e.g., against Dengue virus. This comprehensive review synthesizes current knowledge of miRNA in host response to infection with emphasis on potential clinical applications, along with an evaluation of the challenges still to be overcome.

## Introduction

In 1993, Ambros et al. made a surprise discovery while trying to unpick the fundamental mechanisms underlying gene expression in the nematode. They discovered a 22 nucleotide RNA sequence that controlled expression of a protein encoding gene (1). Initially thought to be a peculiarity of nematodes, the next decade brought the discovery of hundreds of 20–24 nucleotide RNA molecules in viruses, plants, animals and humans, and what’s more, these small RNA molecules were able to regulate the expression of genes (2). These tiny single-stranded transcripts became known as microRNAs (miRNAs, miRs) and led to a paradigm shift in our understanding of gene regulation. Utilizing the mechanics of the RNA interference pathway, miRNAs bind to complementary sequences in the 3′ untranslated region of messenger RNA transcripts, to prevent translation (3). miRNAs fine tune protein production after a gene has been transcribed. Although the study of miRNA is still in its relative infancy, it is clear that miRNAs are key mediators of gene expression. The miRNA registry, miRbase, lists 2558 human miRNAs, and collectively these miRNAs regulate an estimated 60% of protein-coding genes (4, 5). miRNAs are critical controllers of cell differentiation and functions, mediating a variety of cellular processes including those integral to innate and adaptive immunity (6–15). Unsurprisingly, pathogens have evolved to exploit host miRNAs to subvert the immune response (16–34). Characterization of the physiological roles of miRNAs in immunity has led to the pursuit of miRNA-based tests and treatments, and 24 years after the discovery of the first miRNA, the clinical application of miRNAs in infectious disease is starting to be realized. This review summarizes the role of miRNAs in host response to pathogens and reviews the promising clinical applications of miRNAs in preventing, diagnosing, and treating infections.

## MicroRNAs

**Figure 1:**
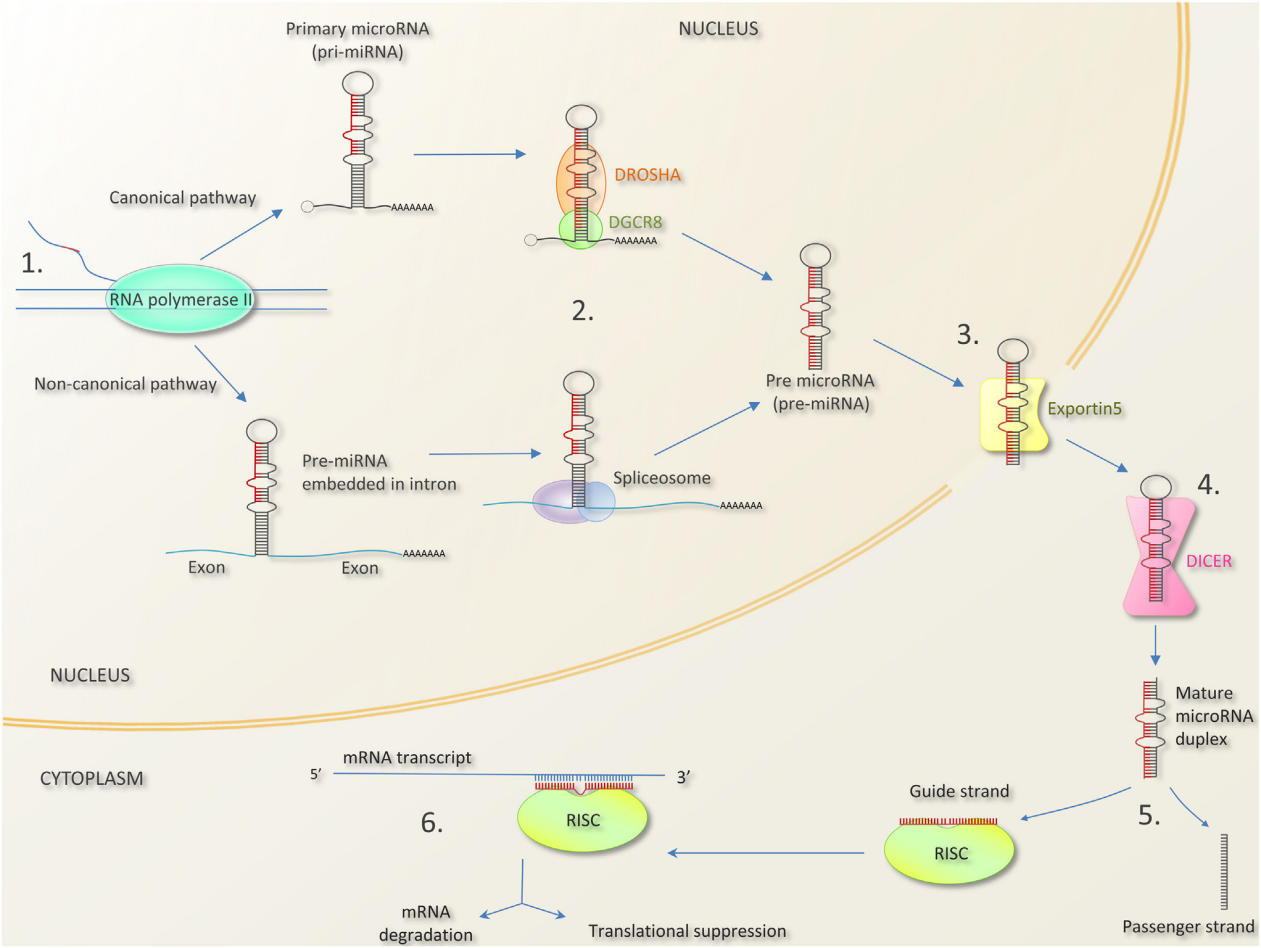
MicroRNA biogenesis and mechanism of action [composed from Ref. (35–39)]. 1. MicroRNA genes can be found as independent transcriptional units, or embedded within introns, and occasionally exons of other genes. In the canonical pathway, microRNA are transcribed as long primary miRNA transcripts (pri-miRNAs) by RNA polymerase II (and occasionally RNA polymerase III). Pri-miRNAs can be several kilobases long and can contain the stem loops of several mature miRNAs (giving rise to miRNA clusters—see Box 1). In the non-canonical pathway, miRNA precursors lie in mRNA introns (“miRtrons”). 2. Pri-miRNAs are processed by the nuclear protein DGCR8 (DiGeorge syndrome critical 8 region) and the enzyme DROSHA into hair pin shaped structures called pre-miRNA transcripts. In the non-canonical pathway, miRNA precursors in mRNA introns are spliced out and bypass DGCR8/DROSHA. 3. Pre-miRNAs are exported to the cytoplasm by exportin-5. 4. The enzyme DICER cleaves the pre-miRNA hairpin loop to produce a mature miRNA duplex. 5. One strand of the miRNA duplex (the guide strand) associates with Argonaut (AGO) protein in the RNA induced silencing complex (RISC). The remaining strand (termed the passenger strand) is degraded. In most cases, there is a preference for which strand is incorporated due to factors like thermodynamic stability. 6. The mature single-stranded miRNA in the miR-RISC complex binds to complementary sequences in the 3′ untranslated region of mRNA molecule, preventing translation. Bound mRNA may be degraded or stored for translation later (48). Bound mRNA may be sequestered into processing bodies (p-bodies) possibly for later release (49).

MicroRNA biogenesis and mechanism of action are summarized in Figure 1 (35–39). Key terminology is outlined in Box 1. As noted above, miRNAs bind to complementary sequences in the 3′ untranslated region of mRNA transcripts. miRNA molecules do not require perfect complementarity to bind mRNA, indeed only nucleotides 2–7 of a miRNA (termed the “seed region”) have to match a site on a mRNA perfectly for binding to occur (40). Therein lies the complexity of miRNA-mediated gene regulation; namely one miRNA can bind hundreds of different mRNAs, and one mRNA can bind several miRNAs, with bound miRNAs cooperatively controlling mRNA transcript translation (41). Generally speaking, binding of miRNA molecules blocks translation and promotes mRNA degradation but this might not always be the case. miRNA binding to 5′ untranslated regions (5′UTRs), exons and even DNA elements has been described and may enhance translation and transcription, respectively (42–47).

Box 1. Terminology ***Nomenclature***. microRNA is commonly condensed to miRNA or miR. Initial miRNAs discovered in *Caenorhabditis elegans* were prefixed with “lin” and “let,” e.g., lin-4 and let-7. Subsequently miRNAs have been numbered in the order in which they were discovered, e.g., miR-1. Their prefix is usually shortened to miR and often prefixed by their species, e.g., hsa-miR-21 (homosapien-microRNA-21).

***−*5p and 3p suffixes:** Denotes whether the mature miRNA is derived from the 5′arm or the 3′arm of the stem loop precursor: e.g., hsa-miR-21-3p, hsa-miR-21-5p.

* ***suffix*:** A * suffix denotes the miRNA strand from the miRNA duplex that is less abundantly expressed as determined by experiment, e.g., miR-56 (the predominant product) and miR-56* (the opposite miRNA strand). This annotation has been generally superseded by the −5p/−3p suffix.

***miRNA family*:** Closely related miRNAs whose mature sequences differ by 1 nucleotide, e.g., the let-7 family, comprising of let-7a, let-7b, let-7c, etc.

***miRNA cluster*:** Some miRNA genes are found grouped together in polycistronic units with a shared promoter meaning they are transcribed together as a long primary transcript and are therefore coexpressed. E.g., miR-17-19 cluster.

***Micronome*:** The entire miRNA expression profile of a cell or sample.

***Seed sequence*:** Nucleotides 2–7 from the 5′ end of the miRNA molecule. Canonical targeting of mRNAs requires that the miRNA seed sequence be perfectly complementary to an mRNA site for binding to occur.

***MicroRNA response element (MRE)*:** the miRNA binding site on an mRNA molecule

***AntagomiR/antimiR*:** A miRNA inhibitor—an artificially produced single-stranded RNA molecule which is perfectly complementary to (and therefore binds to) a target miRNA molecule preventing the miRNA from functioning.

**miRNA mimics:** artificially produced analogs of miRNAs. Used to artificially upregulate the levels of specific miRNAs.

***RNA interference (RNAi), small interfering RNAs (siRNA), and miRNA***.

Small interfering RNAs (siRNAs) are double-stranded RNA molecules which bind to targets with perfectly homologous sequences. MicroRNA and siRNA have analogous but not identical biogenesis. siRNAs are produced from DICER cleavage of endogenously (e.g., mRNA transcript) or exogenously (e.g., viral transcript) derived long double-stranded transcripts. MicroRNA and siRNA pathways conjoin at the point where the guide strand is loaded into RISC complexes (50). Unlike miRNAs, siRNA bind targets with perfect complementarity resulting in mRNA cleavage. In line with several authors this review uses RNAi as an umbrella term for the process of miRNA and siRNA molecules interfering with gene expression. It should be noted that some authors ascribe RNAi solely to the process of siRNA mRNA degradation.

The overall effect of a miRNA on a gene’s protein expression depends on whether its transcript is a direct or indirect target of the miRNA; for example when a miRNA targets a protein’s repressors, that miRNA will indirectly upregulate that protein (the so-called repressor of a repressor effect) (51). The direct and indirect effects of miRNA can therefore lead to protein and pathway repression or enhancement. miRNAs fine tune gene expression often by involvement in complex negative feedback loops and feed-forward mechanisms. (A feedforward mechanism describes a situation where a protein coding transcript and a miRNA targeting that transcript are simultaneously induced by the same factor; this enables a protein to be expressed but not excessively so.) miRNAs may set an expression level threshold which a transcript must reach before protein translation occurs (i.e., the point at which a transcript is so abundant that the inhibitory effects of miRNAs are overcome and protein translation occurs) (11, 51). miRNAs frequently target several proteins in the same or connected pathways (11, 51).

## MicroRNAs are Important Controllers of Leukocyte Development

Evidence that miRNAs are important in the immune system first arose from mice studies showing that the production of mature B and T lymphocytes is altered by manipulating miRNA expression in hematopeotic stem cells and immature lymphyocytes (7, 8, 10). These early studies revealed that miRNAs are pivotal mediators of lymphocyte development and differentiation. More recent work showed that different stages of T-cell development have characteristic, stage-specific miRNA expression profiles, and cell lineage fate (e.g., CD4+, CD8+) is determined by miRNA expression (52, 53). miRNAs also create thresholds which prevent naive T-cells differentiating into effector cells in the absence of significant T-cell receptor activation. For example, naive T-cells, highly express miR-125b which targets factors that promote differentiation (including interferon gamma, IL2-subunit receptor beta, IL10-subunit receptor alpha, and BLIMP1) and differentiation into an effector T-cell will only occur after a reduction in miR-125b caused by T-cell receptor activation (54). miR-125b, miR-181, miR-146, miR-155, miR-150, miR-21, and the miR 17-19 cluster appear particularly important in regulating T-lymphocyte development (55). A similar set of miRNAs, miR-21, mir-34, mir-125, mir-146, mir-155, mir-150, and mir-181 are important in B-cell development and function (56). These miRNAs govern many aspects of immunity, not just lymphocyte development, and are frequently identified as biomarkers of infection (see Biomarker section and tables in Supplementary Material), reflecting their integral role in immune response.

## MicroRNAs Mediate Innate and Adaptive Immunity

**Figure 2:**
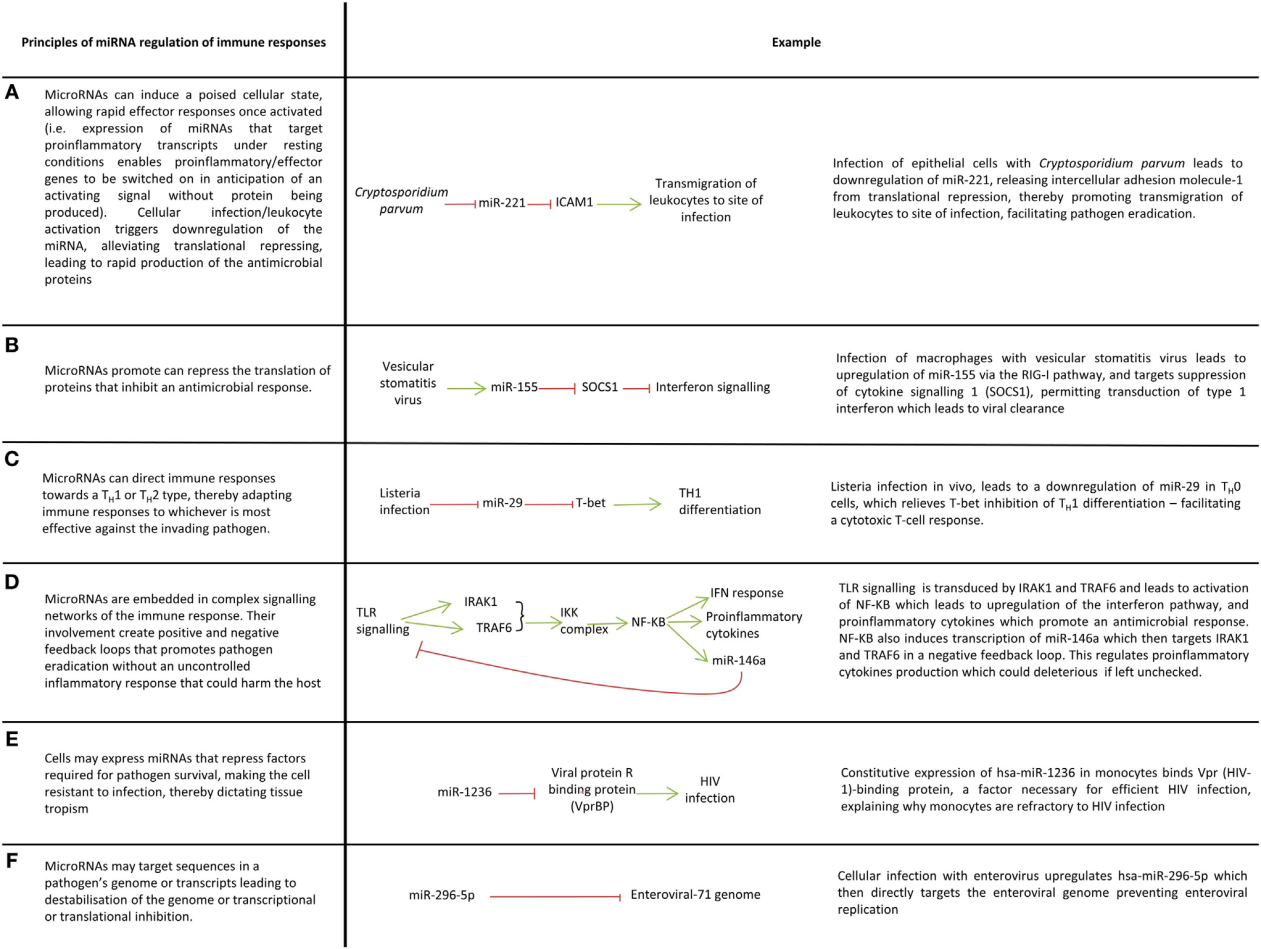
Key ways microRNAs (miRNAs) mediate immune responses to pathogens. **(A–F)** A variety of ways in which miRNAs regulate immune responses.

MicroRNAs have been investigated in the host–pathogen interactions of more than 50 different infections (Table 1). Table S1 in Supplementary Material contains information on which miRNAs have been implicated in these infections along with their proposed targets. Cells isolated from patients infected with viral, fungal, and bacterial infections have different miRNA expression profiles compared with healthy controls, and this is also the case for infected versus non-infected cells *in vitro* (27, 57–62). miRNA regulation has been described in a wide range of leukocytes and in the innate immune responses of non-leukocytes (12, 34, 63–71). Indeed, single nucleotide polymorphisms in miRNA loci have been associated with susceptibility to leprosy and infection outcome in human cytomegalovirus (hCMV) and hepatitis B infection (72–76). Although confirming the functional nature of such associations is difficult, the findings of such studies are in keeping with the important role of miRNAs in immunity and provide some evidence that person to person variation in susceptibility to infection could be governed by polymorphism in miRNA genes, at least for certain pathogens. Key concepts of how miRNAs mediate immunity are illustrated in Figure 2.

**Table 1:** MicroRNAs mediate the host–pathogen interactions of the following pathogens.

Viral infections	Bacterial infections	Fungal infections	Parasitic infections
Chikungunya virus [Selvamani et al. (77)]	*Brucella* spp. [Budak et al. (103)]	*Aspergillus fumigatus* [Dix et al. (116)], *Candida albicans*	*Angiostrongylus cantonensis* [Yu et al. (117)]
**Human Cytomegalovirus** [Hook et al. (78)]	*Francisella tularensis* [Bandyopadhyay et al. (104)]	Muhammad et al. (60)	*Cryptosporidium parvum* [Gong et al. (66)]
Coxsackie virus [Tong et al. (79)]	*Helicobacter pylori* [Teng et al. (105)]		*Leishmania major* [Lemaire et al. (58)]
Dengue virus [Smith et al. (80)]	*Haemophilus influenza* [Tay et al. (106)]		*Malaria falciparum* [Mantel et al. (118)]
Ebola virus [Duy et al. (81)]	*Klebsiella pneumonia* [Teng et al. (71)]		*Schistosomia japonicum* [He et al. (119)]
Epstein–Barr virus [Gao et al. (82)]	*Mycobacterium leprae* [Jorge et al. (107)]		*Toxoplasma gondii* [Cannella et al. (120)]
**Enterovirus 71** [Ho et al. (83)]	*Listeria moncytogenes* [Johnston et al. (108)]		
Hantavirus [Shin et al. (84)]	***Mycobacteria tuberculosis*** [Rothchild et al. (109)]		
**Hepatitis B virus** [Li et al. (85)]	*Neisseria meningitides* [Liu et al. (110)]		
**Hepatitis C virus** [Luna et al. (86)]	*Bordetella pertussis* [Ge et al. (111)]		
Herpes Simplex 1 virus [Pan et al. (87)]	***Salmonella enterica*** [Maudet et al. (27)]		
**Human immunodeficiency virus** [Xu et al. (88)]	*Staphylococcus aureus* [Jin et al. (112)]		
Human papilloma virus [Harden et al. (89)]	*Orientia tsutsugamushi* [Tsai et al. (113)]		
Rotavirus [Chanda et al. (90)]	*Streptococcus pneumoniae* [Griss et al. (114)]		
Human T-cell leukemic virus 1 [Bai and Nicot (91)]	*Haemophilus influenzae* [Tay et al. (106)]		
**Influenza** virus [Tambyah et al. (92)]	*Burkholderia pseudomallei* [Fang et al. (115)]		
Japanese encephalitis virus [Zhu et al. (93)]			
Kaposi’s sarcoma-associated herpes virus [Lagos et al. (94)]			
Polio virus [Perwitasari et al. (95)]			
BK Polyoma virus [Broekema and Imperiale (96)]			
JC Polyoma virus [Rocca et al. (97)]			
Rabies virus [Shi et al. (98)]			
Respiratory syncytial virus [Thornburg et al. (99)]			
Vaccinia virus [Grinberg et al. (22)]			
Varicella zoster virus [Qi et al. (100)]			
West Nile virus [Chugh et al. (101)]			
Zika virus [Pylro et al. (102)]			

*The most frequently studied organisms are highlighted in bold*.

The activation of innate immune cells such as macrophages, dendritic cells, and natural killer cells are controlled by miRNAs (14, 50, 121–125). For example, dendritic cells exposed to mycobacteria, gram-negative and gram-positive bacteria downregulate miR-155, miR-505, miR-7, and miR-940 which alleviates translational repression of proteins involved in innate immune responses (126). miRNAs allow cells to respond rapidly to their surroundings for example, activation of resting NK-cells leads to downregulation of miR-30e which derepresses its target perforin thus enabling prompt cytolysis of infected cells (127). In this way, miRNAs prevent aberrant expression of proinflammatory molecules in the absence of inflammatory stimuli (Figure 2A). miRNAs can also promote inflammatory responses by inhibiting the translation of genes that supress inflammation (Figure 2B).

The plethora of pathogens faced by hosts demands a nuanced immune response which can adapt to cope with viral, bacterial, fungal and protozoal infections. miRNAs facilitate this nuanced inflammatory response through their control of macrophage polarization and T-cell differentiation, thereby mediating the skewing of T-cell immunity toward a T-helper (TH) 1 or TH2 response (Figure 2C). miR-29 for example limits TH1 cell differentiation by targeting mRNAs encoding T-bet, eomesodermin and IFN-gamma which promote TH1 differentiation (15).

A balanced immune response is essential if pathogens are to be eradicated with minimal collateral damage to the host otherwise immune dysregulation as seen in sepsis or chronic inflammation ensues leading to protracted illness and even death. The balance that miRNA provides is best illustrated by their regulation of toll-like receptor (TLR) signaling. TLR signaling induces miRNAs which target many elements of TLR signaling pathways including TLRs themselves, signaling proteins, regulatory molecules, transcription factors and cytokines (6, 9, 13, 125, 128–130). This creates complex feedforward and feedback loops which enables fine-tuning of TLR signaling to ensure pathogens are irradiated in a controlled manner (13) (Figure 2D). miRNA regulation of TLR signaling is a large subject which has been reviewed elsewhere (131) and therefore an extensive discussion is beyond the scope of this review, nevertheless it is useful to briefly mention miR-155 and miR-146 as these are frequently identified in studies investigating host–pathogen interactions. miR-155 is induced by TLR-2, TLR-3, TLR-4, and TLR-9 signaling in many cell types and generally functions as a proinflammatory miRNA through its targeting of factors which negatively regulate inflammation, e.g., SH2-containing inositol 5′-phosphatase 1, suppression of cytokine signaling 1 (13, 130, 132). To prevent excessive inflammation however, miR-155 expression must be controlled. In macrophages, TLR-4 signaling induces both miR-155 and IL-10, an anti-inflammatory cytokine that inhibits transcription of miR-155; this system ensures a check in the upregulation of miR-155 and therefore the inflammation it induces (133). miRNAs also negatively regulated inflammation and miR-146a is a prime example. miR-146a negatively regulates TLR signaling through a negative feedback loop involving its targets IRAK1 and TRAF6 which ensures a check in the proinflammatory signaling of NF-κβ (124, 125).

Although miRNAs such as miR-155 and miR-146a are generally seen as positive and negative regulators of inflammation, respectively, it should be remembered that most miRNAs are likely to be pleiotropic in infection, with net result dependant on the transcription factors and signaling molecules which are concurrently shaping cell response and cell differentiation. miR-155, for example, may promote inflammation as noted above, but in some circumstances it may also negatively regulate NF-κβ activation thereby controlling inflammation (134). The targets of a miRNA can differ from cell type to cell type so the action of a miRNA in a macrophage for example may be different to its action in a neutrophil, or TH1 lymphocyte; care should be taken to avoid generalizing the findings from one cell type to another and it would be a gross oversimplification to classify most miRNAs as proinflammatory or anti-inflammatory (109). Care should also be taken in generalizing the results of *in vitro* experiments to the *in vivo* setting, and in generalizing mice studies to humans as the micronome and targets of miRNA can vary *in vitro* compared to *in vivo* and can be organism specific (135).

Another process tight controlled by of miRNAs is the production of high affinity antibodies. During infection/postvaccination, B-cells, T-follicular helper cells, and dendritic cells form germinal centers in lymphoid tissue. miRNAs are integral to this antibody affinity maturation process, evidenced by studies showing germinal center formation does not occur if there is global knockout out of miRNAs in B-cells (through conditional knock out of DICER) prevents germinal center formation. Within these germinal centers, activated B-cells undergo clonal expansion and mutate their B-cell receptors (somatic hypermutation) which are then tested against antigen bound by follicular dendritic cells in the presence of T-follicular helper cells. B-cells enter apoptosis unless their B-cell receptor strongly binds antigen in which case they receive cell survival signals from T-follicular helper cells which causes upregulation of miR-155 which then targets proapoptotic factors (e.g., JARID2) and reverses the apoptotic pathway (136). Surviving B-cells may undergo further rounds of clonal expansion, somatic hypermutation and antigen presentation; a process which ultimately yields a population of B-cells with high affinity antibodies.

Mutation of the B-cell receptor (through somatic hypermutation) and immunoglobulin class switching is regulated by miRNAs. Activation-induced cytidine deaminase (AID) catalyzes mutation of the immunoglobulin locus and is integral to class switching and affinity maturation but must be tightly controlled to prevent off target mutations and an overzealous mutational rate that can lead to oncogenic mutations and, for unclear reasons, low affinity and autoreactive antibodies (137–139). AID transcripts are targeted in resting B-cells by high levels of miR-181b. miR-181b is downregulated after activation, allowing AID expression and thus class switching and somatic hypermutation. To tightly regulate AID expression, B-cell receptor signaling induces miR-155 (which targets AID transcripts) at the same time as AID (65, 138, 140). Coinduction of AID and miR-155 after B-cell receptor stimulation creates a system in which AID is rapidly induced and then rapidly brought undercontrol, preventing immune pathology (11, 138).

Given the pivotal role of miR-155 in the germinal center response, dysregulation of miR-155 could lead to immune dysfunction in humans; supporting this is the finding that naive B-cells of elderly people have higher levels of mir-155 compared with young people, and this inhibits class switching in the B-cell of elderly people due to increased downregulation of AID. These findings indicate that miRNA modulation of immunity is a finely balanced process and increased susceptibility to infection and possibly poor vaccine responses in elderly people may in part be due to age-related dysregulation of miRNAs (141, 142). Such a conclusion is further supported by a study showing an age-associated decline in miR-125b expression in monocytes and naive CD8+ T-cell correlates with an age-associated increase in expression of its target chemokine (C-C motif) ligand 4 (CCL4) (143). CCL4 is a chemokine that promotes leukocyte migration, activation and T-cell differentiation, and its aberrant over expression in elderly people is thought to contribute to a chronic inflammatory state, leading the authors to suggest that changes in miR-125b with age may underlie age-associated chronic inflammation.

As well as shaping cell response to infection, miRNAs may play a role in determining the tropism of viruses and intracellular bacteria (23, 27, 91, 144) (Figure 2E). Primary monocytes for example are resistant to HIV infection, and this appears to be due to endogenous expression of miR-1236 which targets Vpr (HIV-1)-binding protein (144).

## Host miRNAs May Target Pathogens Directly

In addition to regulating leukocyte response, miRNAs themselves may be independent effectors of innate immunity by directly targeting viral transcripts (see Table S1 in Supplementary Material). *In vitro* studies show miRNA target influenza; vesicular stomatitis virus, human T-cell leukemia virus 1; human papilloma virus; and enterovirus 71, and inhibit viral replication (Figure 2F) facilitating pathogen clearance or potentiating viral latency (91, 145–149). Malaria transcripts have also shown to be targeted by host miRNAs translocating into the parasite (150). Indeed, miRNAs may partially dictate the cell tropism of a virus due to their targeting of viral transcripts, e.g., the resistance of resting T-cells to human T-cell leukemia virus appears to be due to their expression of mir-28-3p (91). Not everyone agrees that miRNA directly target viral transcripts, Bogerd et al. argue that cellular miRNAs do not target viruses as global downregulation of host cell miRNAs (*via* DICER knockout) did not lead to enhancement of 11 viruses in human embryonic kidney cell line 293T (151). Bogerd et al.’s model is problematic however as viruses may be dependent on cell mechanisms which are controlled by miRNAs and also the usual host cell of the viruses in their study are not human embryonic kidney cells. Contrary to Bogerd et al.’s study, there is evidence that direct targeting of viral genome/transcripts occurs *in vivo* as several groups have successfully attenuated viral vaccines by incorporating human miRNA seed sites in viral genome (see The Clinical Applications of miRNAs: Improving Vaccines) (152). The relative importance of miRNA direct targeting of viruses in innate immunity remains to be seen however as *in vivo* and *in vitro* studies show viral mutation of miRNA seed sites in viral genomes means viruses can quickly evolve to avoid being targeted by miRNAs (149, 153).

## Extracellular miRNAs and Host Response to Pathogens

MicroRNAs secreted from cells are called extracellular miRNAs (ex-miRNAs, circulating miRNAs). Extracellular miRNAs can be isolated from most biological fluids (154–156). Ex-miRNAs are contained in extracellular vesicles (exosomes, microvesicles, and apoptotic bodies), and through their association with Argonaut protein (a component of the RISC complex—see Figure 1) and high-density lipoprotein (154, 157–159). The biological function of ex-miRNAs is debated; they may be actively secreted as intercellular communicators of gene regulation; actively secreted as a cellular waste disposal method; or passively secreted as a by-product of cell death (160). Although all three theories may be correct there is increasing evidence that ex-miRNA are functional, can be passed between leukocytes *in vitro* and *in vivo*, and play a role in disease (161–164). Supporting a functional role is the finding that, miR-233 and miR-29a are upregulated in the serum, and unstimulated PBMCs of HIV-exposed seronegative individuals (HESN) compared with healthy controls (165). PBMCs from HESN individuals release greater amounts of miR-223 and miR-29a when infected with HIV *in vitro* and infection is also better controlled, prompting the study’s authors to speculate that miR-223 and miR-29a could represent novel therapeutic targets. Ex-miRNAs have been implicated in the pathophysiology of infection, for example *Malaria falciparum* infected erythrocytes secrete extracellular vesicles containing miR-451a which are taken up by endothelial cells and downregulate proteins required to maintain integrity of the endothelium again suggesting that miRNA-based therapies could hold promise (118) (this issues of this are discussed in the section below on treatments). A key issue for establishing the role of ex-miRNA in infectious disease is identifying the donor and recipient cells of miRNA. The surface marker proteins of exosomes (e.g., CD44) may help in this respect, but miRNA bound to protein and HDL is more difficult to track (166). Regardless of what they do, one clinical application of ex-miRNA is the use of them biomarkers of infectious disease. This is discussed in detail in the Biomarker section.

## Intracellular Pathogens Exploit Host miRNAs

**Figure 3:**
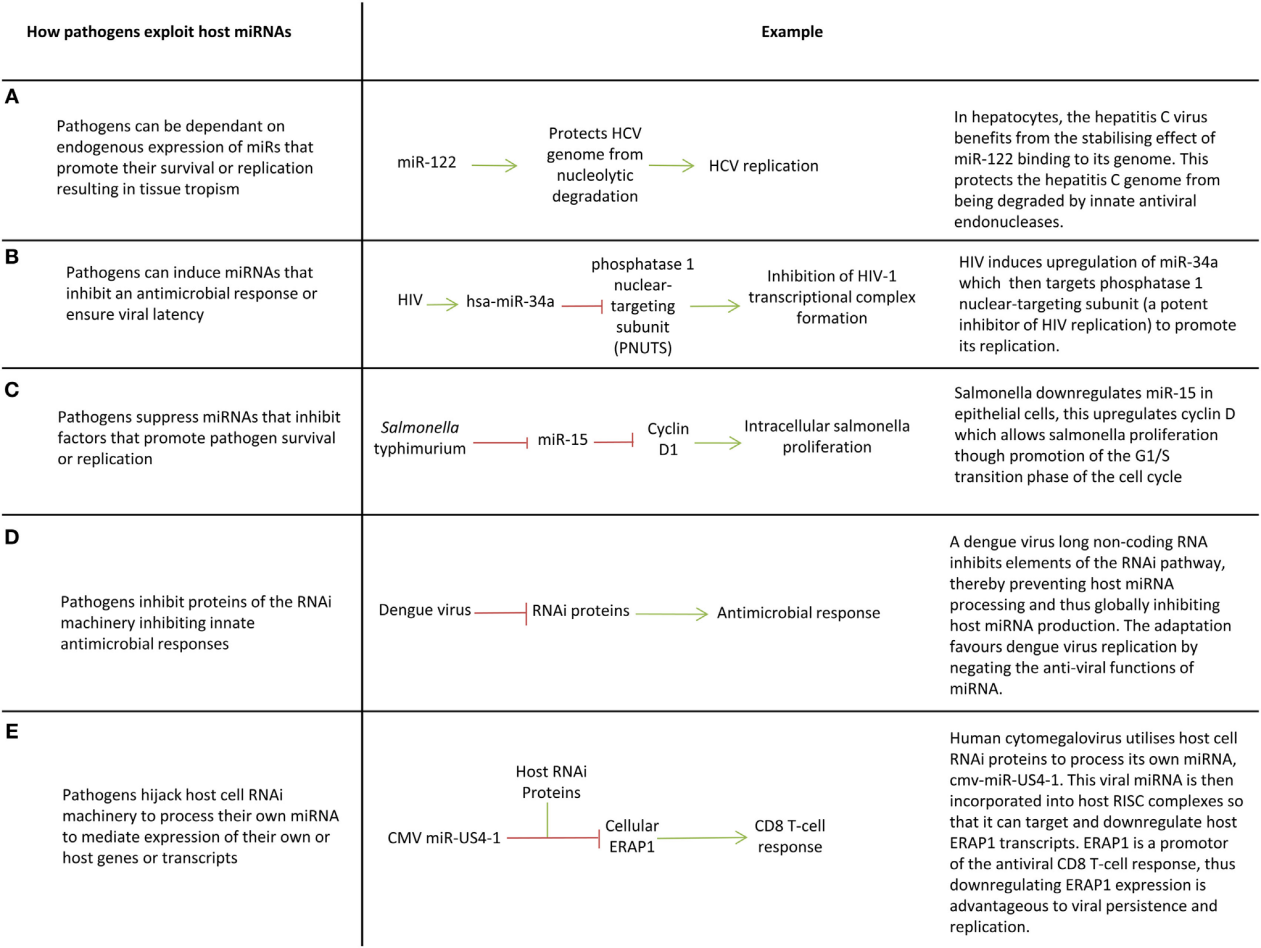
Pathogen exploitation of host microRNAs (miRNAs) and miRNA processing pathways. **(A–E)** Key ways pathogens exploit host miRNAs.

Since miRNAs are part of an effective immunological response, intracellular pathogens have evolved ways of utilizing host miRNAs to create an immune tolerant environment that promotes pathogen survival and latency (Figure 3) (19). One of the first examples of this was the discovery that the hepatitis C virus (HCV) is restricted to hepatocytes because it depends on expression of miR-122 (a liver specific miRNA) to survive and replicate (23) (Figure 3A). miR-122 binds to the HCV genome in two places, including the 5′UTR and this creates a 3′ overhang which protects the virus from nuclear degradation (167, 168). Cellular miRNAs can also promote viral latency. For example, during latent hCMV infection, host miRNAs miR-200c and miR-200b target the transcript of a hCMV protein (UL122) which promotes the lytic replication, thereby promoting latency, and allowing it to persist within the host (169). The switch from viral latency to reactivation can also be facilitated by miRNAs targeting viral transcripts which inhibit lytic replication (20).

Viruses such as enterovirus, CMV, human papilloma virus, hepatitis B virus (HBV), and HCV, manipulate the expression of specific cellular miRNAs to inhibit translation of proinflammatory proteins (16, 17, 21, 24, 25, 27, 29, 32, 33) (Figures 3B,C). For example, Epstein–Barr virus latent membrane protein 1 induces miR-146a expression to negatively regulate the interferon response and promote its survival (17). Manipulation of host miRNAs can occur indirectly through induction or repression of transcription factors, or in some cases may occur directly through viral sequestration of host miRNAs. In the latter case, viral transcripts bind cellular miRNAs through the production of small RNA molecules which are complementary to miRNAs (essentially virally produced antagomiRs) or through multiple cognate miRNA binding sites in miRNA decay elements or long 3′UTRs (miRNA sponge effect) (86, 131, 170–172). This sequestration blocks miRNA function and may promote miRNA decay, preventing miRNAs downregulating host transcripts that are advantageous to viral survival or replication.

There is evidence that some viruses such as HIV, dengue, vaccinia, Epstein–Barr virus, Human papilloma virus 16, and HBV globally suppress miRNA production in host cells to circumnavigate miRNA-mediated innate immunity by targeting proteins involved in the RNA interference pathway (e.g., DICER) (18, 22, 26, 28, 31, 173) (Figure 3D). Given miRNAs mediate host response to infection, it follows that miRNA deregulation could contribute to susceptibility to disease and immunopathology. Studies relating miRNA expression to patient outcomes in infection are now needed to understand how deregulation of miRNAs can contribute to disease course. This could provide new therapeutic targets or prognostic indicators.

Intracellular bacteria including *Mycobacterium* spp., *Listeria monocytogenes, Francisella tularensis*, and *Salmonella enterica* also appear to manipulate host cell miRNA expression to downregulate inflammatory cytokines and the factors and pathways that promote autophagy and cell apoptosis (27, 30, 34, 174). For example, miR-26a is downregulated in macrophages by *Mycobacterium tuberculosis* which leads to depression of the miR-26a target Krüpple-like factor 4 (KLF4). KLF4 is a transcription factor that skews macrophage polarization away from the inflammatory M1 phenotype toward an anti-inflammatory M2 in which bactericidal mechanisms are repressed. Downregulation of miR-26a in *M. tuberculosis* infected cells therefore promotes M2 polarization which facilitates *M. tuberculosis* survival (175). The molecular mechanisms whereby intracellular bacteria manipulate host cell miRNAs remains to be elucidated.

## Viruses have miRNAs Too

Viruses encode miRNAs within in their genome which promote viral replication and control latency (96, 176–183). HIV, Ebola, Adenoviridae, Herpesviridae, and the BK polyomavirus utilize host cell machinery to process their own miRNAs (96, 176–183) (Figure 3E). Viral miRNAs promote viral latency, prevent cell apoptosis (an oncogenic feature of oncoviruses such as Kaposi’s sarcoma-associated herpes virus that promotes malignant transformation), and downregulate factors which promote an inflammatory response and recruitment of leukocytes (184). The HSV-1 gene latency-associated transcript, for example, encodes miR-H2-3p which is cotranscribed with, and targets a viral immediate-early transcriptional activator (ICPO) believed to promote HSV-1 replication and reactivation from latency (183). CMV inhibits CD8(+) T-cell responses by expressing miR-US4-1 which targets endoplasmic aminopeptidase 1, a protein responsible for trimming peptides for presentation by MHC class I molecules (179). The Kaposi’s sarcoma-associated virus expresses many miRNAs including miR-K5 and miR-K9 which target MYD88 and IRAK1 to reduce inflammatory cytokine expression and clearance by the immune system (19). Viral miRNAs may mimic host miRNAs, e.g., Ebola virus and the Kaposi’s sarcoma-associated herpes virus encode miRNA orthologs of miR-155 (180, 185). In the case of Ebola, this miRNA-155 analog targets importin-α5 (KPNA1) expression which may lead to dysfunction of the interferon signaling and an unbalanced immune response thereby contributing to the marked pathogenicity of the virus (180). Manipulating viral miRNAs using miRNA mimics or antagomiRs could hold therapeutic potential. Several studies show viral miRNAs are excreted in exosomes of infected cells, and in some cases this appears to modulate innate immune responses in recipient cells including dendritic cells and macrophages; however, the functional significance of this remains to be seen (186–190).

There is little evidence that bacteria have miRNAs. Deep sequencing of a mouse macrophage cell line (RAW264.7) infected with *Mycobacterium marinum* identified a 23nt RNA molecule which is predicted to arise from a stem loop, and was not present in broth grown bacteria, prompting the authors to suggest DICER in the host cell is required for processing as would be expected for a miRNA (191). Evidence is far from conclusive however and deep sequencing of human cells infected with *Chlamydia trachomatis, Legionella pneumophila*, and *M. tuberculosis* in the same study did not identify any potential bacterial miRNAs.

## The Clinical Applications of miRNAs: Treatments

A promising application of miRNAs is to utilize their immunomodulatory functions to promote antimicrobial pathways during infection and control dysregulated inflammatory responses during sepsis. Hock et al., noted physiological downregulation of miR-328-3p in the lungs of mice infected with non-typeable *Haemophilus influenza* promoted phagocytosis by neutrophils and macrophages and bacterial killing and found boosting this downregulation by intra-tracheally administering an antagomiR (see Box 1) of miR-328-3p enhanced bacterial killing when mice were challenged with non-typeable *H. influenza*. Alexander et al. observed that administration of exosomes containing miR-146a and miR-155 ameliorated and enhanced mice inflammatory response to endotoxin *in vivo*, respectively, prompting the authors to speculate that such treatments could be useful adjuncts in managing sepsis (in the case of miR-146a) or vaccination (in the case of miR-155). Work by Wang et al. supports the notion that miRNA therapies could be a useful treatment in sepsis after they discovered administration of mesenchymal stem cell exosomes containing miR-223 confer cardiac protection in septic mice (192).

A miRNA-based strategy that renders the host resistant to the exploitation of their miRNAs by pathogens opens up a new avenue of therapeutics. As noted above, EV-71 infection induces miRNA-146a in cells to prevent an innate immune response. Work by Ho et al. showed that inhibition of miR-146a in EV-71 infected mice using an intraperitoneal injection of a miR-146a antagomiR significantly improved survival from 25 to 80% by reinstating an interferon gamma response (83). Bacterial infections may also be treatable with miRNAs. Upregulation of miR-128 by *S. enterica* promoted *S. enterica* survival in mice and intragastric delivery of anti-miR-128 reverses this phenomenon in mice and suppressed *S. enterica* infection (193).

Translation of these concepts to clinical treatments is on the horizon. An oligonucleotide inhibitor of miR-122 called Miravirsen was shown to be proven to be safe and well tolerated in HCV-infected patients in a phase IIa trial (194). In an effort to target drug delivery more effectively RG-101 was created in which the oligonucleotide inhibitor is conjugated to a high affinity ligand for the hepatocyte-specific asialoglycoprotein receptor (ASGPR). A recent phase 1b double blind randomized controlled trial of RG-101 showed a single subcutaneous dose induced a significant reduction in viral load in patients with HCV within 4 weeks of treatment (195). Unfortunately, viral rebound was seen in most patients (22 out of 28) and this was associated with HCV 5′UTR mutations. A small number of patients (3 out 28) had a sustained antiviral response at 76 weeks and phase II trials are planned to see whether combining RG-101 with virus targeting antiviral agents augments HCV therapy. Interestingly RG-101 appeared to increase NK-cell frequency and reduce activation which may have contributed to control of HCV (196). The hope is a treatment like RG-101 could shorten current HCV treatment regimens or offer an alternative treatment option in patients who have not responded to standard therapies (195).

Overall, using miRNA-based therapies to leverage immune response may prove useful adjuncts to standard antimicrobial therapies, e.g., in multidrug resistant gram-negative infections, or chronic viral infections such as hepatitis C. Nevertheless, there are significant challenges to implementing miRNA-based antimicrobial therapeutics which include devising methods of administration and drug design that will protect miRNA mimics/antagomiRs from circulating RNAse enzymes. Delivery systems have to ensure targeted efficient delivery of miRNAs to the site of infection, because, as noted above, a miRNA may appear in many cell types, serving very different functions making off target effects a real possibility, limiting efficacy and safety (134). A detailed analysis of the outcomes of phase 1 trials of two miRNA-based cancer treatments will provide more important data on the feasibility of miRNA-based therapeutics generally (197, 198). As noted in the RG-101 trial above, viral mutation and resistance is an issue that will need tackling (195).

## The Clinical Applications of miRNAs: Biomarkers of Infectious Disease

Extracellular miRNAs are ideal biomarker candidates because they can be isolated from biological fluids (199). RT-PCR is already used routinely in the clinical setting to quickly identify infections (e.g., respiratory infections in babies with bronchiolitis) and could be used to quantify ex-miRNAs in patient samples.

A comprehensive search of the literature identified 57 studies assessing ex-miRNAs in infectious diseases through whole micronome profiling and candidate miRNA approaches (see Tables S2 and S3 in Supplementary Material). The overwhelming majority of these studies are serum and plasma based, but ex-miRNAs in CSF, saliva and sputum have also been investigated. Most studies, to date, have focused on HCV, HBV, HIV, tuberculosis (TB), and sepsis and aim to improve diagnosis and prognosticate infection outcome (e.g., death in sepsis, liver cirrhosis in hepatitis) or treatment response.

Most infection studies compare the ex-miRNA profile of patients with healthy controls. Many identify ex-miRNA signatures which are highly predictive of infection. Zhang et al. found the combined expression of miR-378, miR-483-5p, miR-22, miR-29c, miR-101, and miR-320b could differentiate pulmonary TB from healthy controls with sensitivity and specificity of 95 and 91.8%, respectively (200). The issue with using healthy controls as a comparator group is that differentially expressed ex-miRNAs may represent a non-specific marker of infection; this limits the clinical translatability of these studies given most people undergoing tests are symptomatic of some disease process.

A handful of studies have chosen more pragmatic comparator groups and promisingly suggest ex-miRNA signatures can differentiate particular infectious disease from other differential diagnoses. Zhang et al. found miR-379, 483-5p, 23, 29c were upregulated, and miRNA 102 and 320b were downregulated in the serum of patients with pulmonary TB compared to patients with pneumonia, lung cancer, and COPD and this was confirmed in a second independent cohort (200). Interesting data from investigators in China identified miRNA signatures which differentiate enteroviral hand foot and mouth, coxsackie hand foot and mouth, pertussis, TB, and varicella from each other (100, 201).

A promising application of ex-miRNA biomarker work may be to differentiate viral from bacterial infection, identify or prognosticate sepsis, and in monitoring of response to antimicrobial treatment. At least 13 studies investigating ex-miRNA signatures in sepsis have been published; most are conducted in an intensive care unit (ICU) setting. In more than one study, miR-223, miR-193, miR-483, miR-499, miR-15a/b, and miR-16 have been identified as potential biomarkers of sepsis diagnosis and mortality (see Tables S2 and S3 in Supplementary Material). There are substantial inter-study discrepancies in miRNAs identified as potential biomarkers; this may be due heterogeneity in study design (e.g., data normalization methods) and confounders such as hospital differences in defining sepsis and ICU admission criteria. Differences in the lengths of illness between patients creates noise in the data; longitudinal studies that measure serial miRNA levels would provide temporal information on miRNA expression in sepsis and may resolve some conflicting findings.

The most commonly identified biomarker across all ex-miRNA biomarkers of infection studies is miR-122 because of the over-representation of hepatitis studies in ex-miRNA biomarker studies. Studies of hepatitis C and hepatitis B have repeatedly found an association between circulating miR-122 and an aspect of infection (202–204). miR-122 is highly expressed by hepatocytes, and the association between levels of serum miR-122 and hepatitis may be the result of hepatocyte death. Upregulation of serum miR-122 is associated with HCV and HBV infection *per se*, correlates with viral DNA titers and falls during antiviral treatment (202–204). Conflicting evidence exists regarding the use of baseline miR-122 to prognosticate likelihood of response to treatment and liver cirrhosis (202–205). Among the 30 biomarker studies which used a miRNA profiling method (rather than a candidate miRNA approach which are at risk of heavy reporting bias), the most commonly identified ex-miRNAs biomarkers were miR-122 and the miR-29 family (identified in 7 studies), miR-21 and miR-146 (identified in 4 studies), and miR-150, miR-16, miR-22, miR-125, miR-134, miR-194, and miR-106 (identified in 3 studies). Given the significant heterogeneity across all studies, there are significant limitations in combining them in this way, nevertheless it is interesting to note that a several of these miRNAs (miR-125, miR-150, and miR-21) already have established roles in lymphocyte development and activation (54, 56).

There are challenges in using miRNAs as biomarkers of infectious disease, however, and this is underlined by a lack of inter-study cross validation of many results. Conflicting study results may arise from heterogeneity in study design including differences in populations and control groups; methods of miRNA extraction and the circulating fraction under investigation (serum, plasma, microvesicles, or exosomes); micronome expression profiling platforms (next generation sequencing, probe-based hybridization microarrays, or RT-PCR arrays) and the dearth of miRNAs assessed; the limited statistical power of many studies at the profiling stage; data normalization methods; whether p-values were adjusted to take account of multiple testing issues (usually not done); and whether confirmatory cohorts were used to validate results. Given there is good evidence that miRNA contained in exosomes is functionally secreted as intercellular mediators of gene regulation, it is tempting to speculate that biomarker studies which profile miRNAs in exosomes rather than ex-miRNA in total plasma/serum (which will include a background of miRNA present from dead cells) could be more sensitive or specific biomarkers; comparisons of different extraction methods within the same biomarker study could help resolve this possibility.

## The Clinical Applications of miRNAs: Improving Vaccines

**Figure 4:**
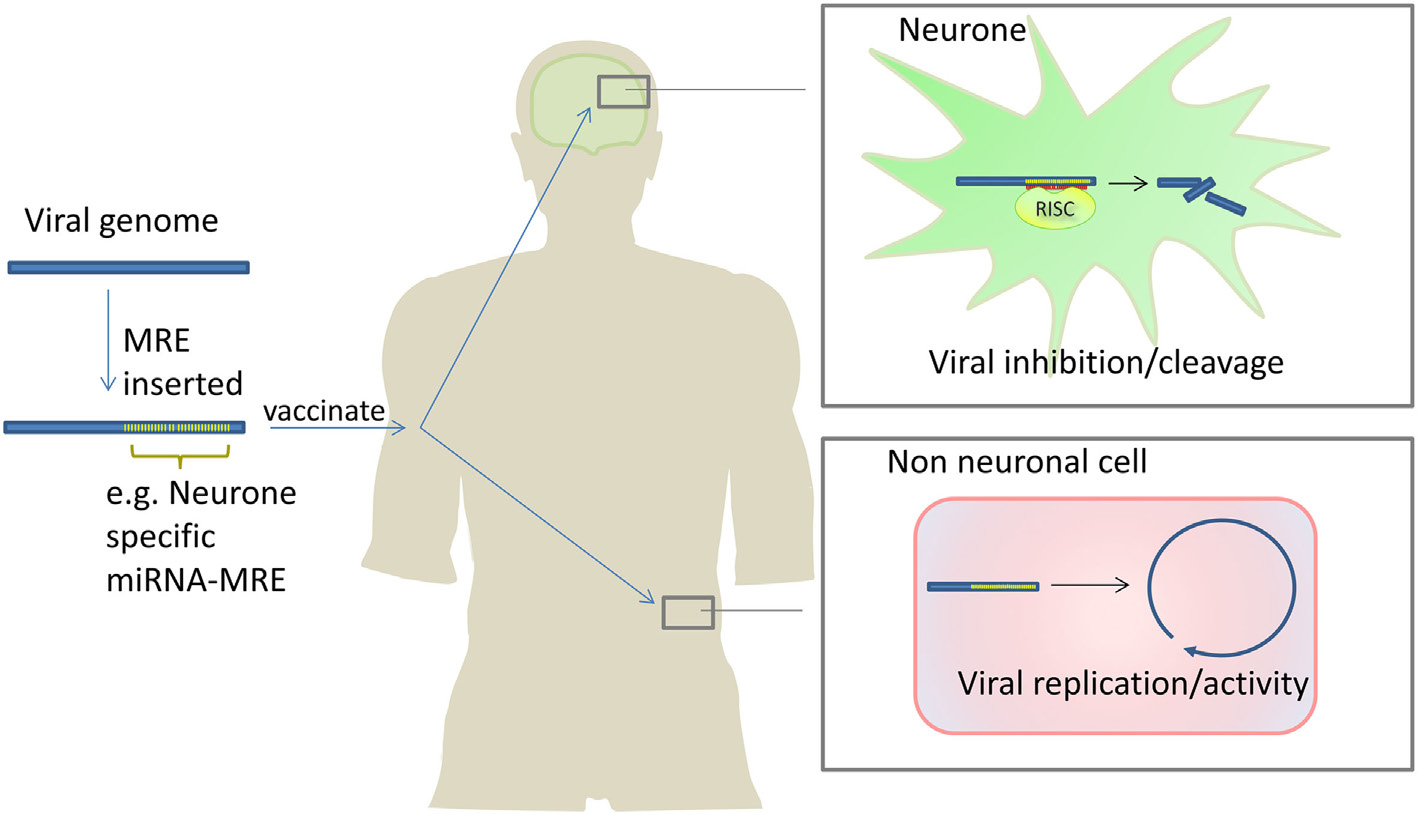
Attenuating live viral vaccines by inclusion of microRNA response elements (MREs). Insertion of MREs in a viral genome can make viral vaccines safer because the virus is unable to replicate in cells which express the miRNAs which bind to the inserted MREs. In this example, a neurone-specific miRNA MRE is inserted into a virus which attacks the nervous system. The cognate miRNA is expressed in neurones inhibiting viral replication/activity. In non-neuronal cells, the miRNA is not expressed therefore the virus is able to replicate and infect cells stimulating robust immunity. Barnes et al. have used this approach to develop an attenuated polio vaccine in mice (152).

As previously discussed, miRNAs may inhibit viruses through direct targeting of viral genomes or transcripts. This concept is being exploited to create new and attenuated vaccines by incorporating miRNA response elements (miRNA target sequences) into viral vaccine genomes. When the virus enters a cell-type which expresses the miRNA which targets the miRNA response elements (MRE), the virus is attenuated. The virus is not attenuated in other cells not expressing that miRNA. In theory, this allows the creation of live vaccines which are attenuated in a tissue specific manner, maximizing efficacy while minimizing harm (see Figure 4).

Several groups have demonstrated safe, efficacious vaccination in mice through the incorporation of MREs into viral genomes. Perez et al. incorporated the ubiquitously expressed miR-93 MRE into a live influenza virus and showed the vaccine was safe and effective in mice (206). Most groups use MREs that correspond to tissue-specific miRNAs; an example being the incorporation of miR-124 in polio, West Nile, and Dengue viruses to selectively prevent replication in neuronal cells; and the incorporation of a let-7 MREs in H1N1 viral vaccines to reduce replication in bronchial cells (152, 207–210). In all of these cases, vaccination was efficacious and viruses were significantly attenuated. In two studies, a small number of immunocompromised mice succumbed to infection, and this was due to MRE mutation (152, 153). Increasing the number of MREs repeats, adding MREs at different sites, incorporating multiple, different MREs in the viral genome, or combining MREs with other genome alterations that attenuate viruses has been shown to ameliorate this (153, 211).

MicroRNA could also be used as adjuvants to regulate proteins which could inhibit vaccine response. Protein kinase R-like endoplasmic reticulum kinase (PERK) triggers apoptosis in response to viral infection which can inhibit DNA vaccines from working. Wheatley et al. developed a plasmid-based HIV vaccine which expressed HIV-1 envelope (Env) antigens as well as a miRNA designed to inhibit PERK (an *in silico* designed miRNA named miR-huPERK) (212). To make this plasmid the sequence encoding miR-huPERK inserted into the miR-155 gene (the mature miR-155 sequence was removed but the rest of the pri-miR-155 sequence remained) and then cloned into the plasmid along with Env. Transfecting cell lines with this plasmid reduced PERK expression only when the mir-155 gene was used as a scaffold for miR-huPERK. Vaccinating BALB/c mice with this modified DNA vaccine augmented Env-specific CD8+ T-cell immunity. The study provides proof of principle that incorporation of miRNAs into vaccine constructs can ameliorate innate antiviral pathways which usually limit maximal antigen expression; the result is enhanced immunogenicity of DNA vaccines; potentially enabling development of novel DNA vaccines (212).

## Conclusion

MicroRNAs are essential mediators of host response to pathogens. They have pleiotropic roles which microbes have evolved to exploit. Elucidating the roles miRNAs in host response to infectious disease is inherently interesting at it provides a tool for identifying key genes and pathways that must be activated, enhanced, repressed, or silenced to facilitate an effective immune response. The complex regulatory network within which miRNAs are embedded, make unpicking the roles of miRNAs tough but not impossible. Integrating large miRNA and mRNA datasets using advanced statistical techniques (in a “systems biology” approach) will facilitate the unpicking of these complex networks.

Clinical applications of miRNAs are on the horizon. The novel anti-miRNA treatment miravirsen is already in phase 2b trials suggesting miRNA-based treatments may well become a reality; viral vaccines attenuated through incorporation of miRNA target sequences are at the preclinical stage; and miRNA biomarkers of infection hold promise. In all cases however there are challenges that must be overcome. miRNAs have multiple targets and therefore any vaccines or treatments that harness miRNAs may produce off-target effects compromising safety. In the context of ex-miRNA biomarkers identification of universal endogenous controls are needed to improve cross study reproducibility of findings, and a more standardized approach to biomarker studies may also help. Initiatives devoted to harnessing the diagnostic and therapeutic potential of extracellular RNAs like The National Institute for Health Extracellular Communication Consortium can facilitate this (213).

As the literature on miRNAs grows, the potential for new miRNA therapeutics, diagnostics/prognostics, and vaccines becomes tangibly closer. Translating the insights of miRNA studies into improving the lives of patients is the critical next step.

## Author Contributions

RD conceptualized, researched, and wrote the manuscript. DO reviewed the manuscript and made substantial contributions to the drafting process. AP conceptualized, reviewed the manuscript, and made substantial contributions to the drafting process.

## Conflict of Interest Statement

The authors declare that the research was conducted in the absence of any commercial or financial relationships that could be construed as a potential conflict of interest.

## Abbreviations

3′UTR, 3′ untranslated region; 5′UTR, 5′ untranslated region; AID, activation-induced cytidine deaminase; EV-71, enterovirus 71; HBV, hepatitis B virus; HCMV, human cytomegalovirus; HCV, hepatitis C virus; HESN, HIV-exposed seronegative individuals; HSV, herpes simplex virus; miRNA, microRNA; MRE, microRNA response element; RISC, RNA induced silencing complex; siRNA, small interfering RNA; TB, tuberculosis; TLR, toll-like receptor.
