# Supplementary material for: The Clinical Application of MicroRNAs in Infectious Disease
Source: Front Immunol. 2017 Sep 25;8:1182. doi: 10.3389/fimmu.2017.01182 (PMC5622146; doi:10.3389/fimmu.2017.01182)
Supplement: Supplementary file 1 [file Table_1.DOCX]

Supplementary Material: Table 1

**The Clinical Application of MicroRNA in infectious Disease.**

**Authors: Ruth Drury MBChB^1*^, Daniel O’Connor DPhil ^1^, Andrew J Pollard FMedSci^1^**

^1.^ Oxford Vaccine Group, Centre for Clinical Vaccinology and Tropical Medicine, Department of Paediatrics, University of Oxford, The Churchill Hospital, Old Road, Oxford OX3 7LE, UK

**Corresponding Author:** Ruth Drury, Oxford Vaccine Group, Centre for Clinical Vaccinology and Tropical Medicine, Department of Paediatrics, University of Oxford, The Churchill Hospital, Old Road, Oxford OX3 7LJ, UK; email: ruth.drury@paediatrics.ox.ac.uk; Tel: 01865857420.

**Supplementary table 1. Studies of miRNAs in various infections plus proposed targets.**

It should be noted that each miRNA likely regulates many mRNAs, not just the one identified by the studies. It should also be noted that differentiating direct targets (miRNA binds directly to the target) and indirect targets (miRNA regulates a factor which regulates the target) of a miRNA is difficult and not necessarily established across all studies.

| Infection | miRNA | Target | Target’s function | Reference | |
| --- | --- | --- | --- | --- | --- |
| *Burkholderia pseudomallei* | miR-4458 | autophagy-related 10 (ATG10) | Promotes autophagy | (Li et al., 2015c) | |
|  | miR-4667 | autophagy-related 10 (ATG10) | Promotes autophagy |  |  |
|  | miR-4668 | autophagy-related 10 (ATG10) | Promotes autophagy |  |  |
| Chikungunya virus | miR-146a | TNF receptor associated factor 6 (TRAF6) | TLR signalling, promotes antiviral interferon responses | (Selvamani et al., 2014) |  |
|  |  | Interleukin 1 Receptor Associated Kinase 1 (IRAK1) | TLR signalling, promotes antiviral interferon responses |  |  |
|  |  | Interleukin 1 Receptor Associated Kinase 2 (IRAK2) | TLR signalling, promotes antiviral interferon responses |  |  |
| Coxsackie virus | miR-10 | Coxsackie genome |  | (Tong et al., 2013) |  |
|  | miR-126 | Sprouty-related, EVH1 domain-containing protein 1 (SPRED-1) | Negatively regulates factors promoting cell cycle | (Ye et al., 2013) |  |
|  | miR-214 | Itchy E3 Ubiquitin Protein Ligase | Negative regulator of NF-κB | (Chen et al., 2015) |  |
|  | miR-221-222 cluster | Interferon regulatory factor 2 (IRF2) | Negatively regulates transcription of typ1 inteferons | (Corsten et al., 2015) |  |
|  |  | Chemokine (C-X-C motif) ligand 12  CXCL12 | strong lymphocyte attractant |  |  |
|  |  | thymocyte selection-associated high mobility group box protein (TOX) | Regulator of T-cell development, also expressed in cardio myocytes |  |  |
|  | miR-466 | Coxsackie virus and Adenovirus Receptor (CXADR) | Critical for normal heart function but also used by coxsackie and adenovirus to enter cells. | (Lam et al., 2015) |  |
| *Cryptosporidium parvum* | let-7i | Toll like receptor 4 (TLR4) | Recognition of viral and bacterial antigens including lipopolysacheride | (Chen et al., 2007) |  |
|  | let-7 | Src homology 2-containing protein (CIS) | cytokine-inducible  negative regulator for inflammatory cytokine signalling | (Hu et al., 2009) |  |
|  | miR-98 | Src homology 2-containing protein (CIS) |  |  |  |
|  | miR-221 | Intercellular adhesion molecule-1 (ICAM1) | Transmigration of leukocytes into tissues | (Gong et al., 2011) |  |
| Cytomegalovirus | miR-21 | Cell division cycle 25 homolog A (CDC25A) | Cell cycle regulator | (Fu et al., 2015) |  |
|  | miR-132 | Histone acetyltransferase p300 (p300) | transcriptional co-activator | (Lagos et al., 2010) |  |
|  | miR-155 | SH2 domain-containing inositol 5-phosphatase 1 (SHIP1) | Negative regulator of IFN-gamma production | (Sullivan et al., 2013) |  |
|  |  | Lymphocyte cytosolic protein 2 (SLP-76) | Promotes T-cell development and activation |  |  |
|  | miR-376a | HLA class 1 histocompatibility antigen alpha chain E (HLA-E) | Presents peptides to NK cells | (Nachmani et al., 2014) |  |
| Dengue virus | let-7c | Transcription regulator protein BACH1 (BACH1) | Regulate the oxidative stress pathway | (Escalera-Cueto et al., 2015) |  |
|  | miR-133a | polypyrimidine tract binding protein (PTB) | Involved in mRNA processing, required for Dengue replication | (Castillo et al., 2016) |  |
|  | miR-146a | TNFR-associated factor 6 (TRAF6) | Mediates TLR signalling | (Wu et al., 2013) |  |
|  | miR-150 | Suppression of cytokine signalling 1 (SOCS1) | Negatively regulates cytokine signalling | (Chen et al., 2014) |  |
|  | miR-223 | Microtubule-destabilizing protein stathmin 1 (STMN1) | A key microtubule regulatory protein | (Wu et al., 2014) |  |
| Enterovirus | let-7b | Cyclin D1 (CCND1) | Cell cycle regulator | (Du et al., 2015) |  |
|  | miR-23b | Enteroviral VP1 protein | Capsid protein | (Wen et al., 2013) |  |
|  | miR-141 | Eukaryotic translation initiation factor 4E (eIF4E) | Promotes cap dependant translation. Must be switched off to allow translation of viral proteins | (Ho et al., 2011) |  |
|  | miR-146a | Interleukin-1 receptor-associated kinase 1 (IRAK1) TNF receptor associated factor 6 (TRAF6) | Mediate TLR signalling | (Ho et al., 2014) |  |
|  | miR-197 | RAs-related Nuclear protein (RAN) | nucleocytoplasmic transport of the viral and host proteins | (Tang et al., 2016) |  |
|  | miR-296-5p | Enteroviral genome |  | (Zheng et al., 2013) |  |
|  | miR-526a | cylindromatosis (CYLD) | Negatively regulates interferon 1 production by ubiquinating elements of the RIG pathway | (Xu et al., 2014a) |  |
|  | miR-548 | Interleukin-29 (IL-29) | Part of the interferon pathway | (Li et al., 2013c) |  |
|  | miR-1246 | disc-large homolog 3 (DLG3) | Involved in NMDA receptor-mediated signaling and synaptic plasticity | (Xu et al., 2014d) |  |
| Epstein Barr virus | let-7 | Dicer | Integral protein in miRNA processing | (Mansouri et al., 2014) |  |
|  | miR-17/92 cluster | latent viral genes | Essential for maintain viral latency | (Skalsky et al., 2012) |  |
|  | miR-203 | cyclin G1 (CCNG1) | Cell cycle regulator | (Yu et al., 2012) |  |
|  |  | E2F transcription factor 3 (E2F3) | Transcription factor, cell cycle regulator |  |  |
| *Francisella tularensis* | miR-155 | Myeloid Differentiation Primary Response 88 (MyD88) | Adapter molecule involved in toll like receptor signalling | (Bandyopadhyay et al., 2014) |  |
| *Helicobcter pylori* | miR-146b | Interleukin-6 (IL6) | Proinflammatory cytokine | (Cheng et al., 2015a) |  |
|  | let-7b | Toll-like receptor 4 (TLR4) | Recognition of viral and bacterial antigens including lipopolysachheride | (Teng et al., 2013) |  |
|  | miR-155 | protein kinase A inhibitor alpha (PKIalpha) | Inhibits intracellular cAMP production | (Fassi Fehri et al., 2010)(Tang et al., 2010)(Xiao et al., 2009) (Cheng et al., 2015a) |  |
|  |  | myeloid differentiation protein 88 (MyD88) | Toll like receptor signalling |  |  |
|  |  | Mother against decapentaplegic homolog 2 (SMAD2) | primary signaling pathway downstream of  transforming growth factor-  b |  |  |
|  |  | I kappa beta kinase epsilon (IKK-ε) | Regulates NF-κβ activation |  |  |
|  |  | Fas-associated protein with death domain (FADD) | Apoptosis signalling |  |  |
|  |  | Interleukin-6 (IL-6) | Proinflammatory cytokine |  |  |
| Hepatitis B virus | miR-15b | hepatocyte nuclear factor 1α (HNF1α) | negative regulator of HBV Enhancer I | (Dai et al., 2014) |  |
|  | miR-122 | Cytosolic 5’-nucleotidase 3 (NT5C3) | An interferon stimulated gene | (Hao et al., 2013) |  |
|  |  | cyclin G(1) (CCNG1) | Cell cycle regulator | (Wang et al., 2012) |  |
|  |  | Supressor of cytokine signalling (SOCS3) | Negative regulator of cytokine signalling | (Gao et al., 2015) |  |
|  | miR-125a | HBV surface antigen |  | (Mosca et al., 2014) |  |
|  | miR-130a | Peroxisome proliferator-activated receptor gamma coactivator 1-alpha (PGC-1α) | Involved in lipid metabolism | (Huang et al., 2015) |  |
|  |  | Peroxisome proliferator-activated receptor gamma (PPARγ) | lipogenic transcription factor |  |  |
|  | miR-142 | HBV transcripts |  | (Khlaiphuengsin et al., 2015) |  |
|  | miR-146a | signal transducers and activators of transcription1 (STAT1) | Involved in the interferon response | (Hou et al., 2014)(Wang et al., 2013)  (Li et al., 2015b) |  |
|  |  | complement factor H (CFH) | Complement activation against pathogens |  |  |
|  | miR-155 | CCAAT/enhancer-binding protein-β (C/EBP-β) | Transcriptioin factor. Involved in hepatocyte metabolism and proliferation but can bind to the HBV genome to promote transcription | (Sarkar et al., 2015)(Su et al., 2011) |  |
|  | miR-373 | nuclear factor I/B (NFIB) | Cellular transcription factor, binds to sites on the HBV genome | (Guo et al., 2011) |  |
|  | miR-372 | nuclear factor I/B (NFIB) | Cellular transcription factor, binds to sites on the HBV genome | (Guo et al., 2011) |  |
|  | miR-384 | HBV transcripts |  | (Khlaiphuengsin et al., 2015) |  |
|  | miR-500b | HBV transcripts |  | (Khlaiphuengsin et al., 2015) |  |
|  | miR-548 | Interferon gamma receptor 1 (IFNγR1) | Involved in the interferon response | (Xing et al., 2014) |  |
|  | miR-581 | Dicer (DICER1) | Integral protein in miRNA processing | (Wang et al., 2014) |  |
|  |  | Endoplasmic reticulum degradation-enhancing alpha-mannosidase-like protein 1 (EDEM1) | regulates the degradation of HBV envelope proteins |  |  |
|  | miR-1231 | HBV core mRNA |  | (Kohno et al., 2014) |  |
|  | miR-4731 | HBV transcripts |  | (Khlaiphuengsin et al., 2015) |  |
|  | miR-5193 | HBV surface antigen |  | (Khlaiphuengsin et al., 2015) |  |
| Hepatitis C virus | let-7b | HCV genome |  | (Cheng et al., 2012) |  |
|  | let-7s | insulin-like growth factor 2 mRNA-binding protein 1 (IGF2BP1) | Regulates the growth factor IGF2 | (Cheng et al., 2013) |  |
|  | miR-21 | myeloid differentiation factor 88 (MyD88)  Interleukin-1 receptor-associated kinase 1 (IRAK1) | Mediate TLR signalling | (Chen et al., 2013)  (Gelley et al., 2014) |  |
|  | miR-27b | peroxisome proliferator-activated receptor (PPAR-α) | key nuclear receptor that transcriptionally activates genes associated with fatty acid oxidation | (Singaravelu et al., 2014) |  |
|  | miR-107 | Janus Kinase 1 (JAK1) | Signalling molecules of the interferon response | (Sarma et al., 2014) |  |
|  | miR-122 | HCV genome |  | (Fukuhara et al., 2012)  (Jabłonowska et al., 2014)(Jangra et al., 2010)(Jopling et al., 2005) (Li et al., 2013b)(Li et al., 2011)(Luna et al., 2015) (Shan et al., 2007) |  |
|  |  | Suppressor of cytokine signalling 1 (SOCS1) | Negatively regulate cytokine signalling | (Li et al., 2013a) |  |
|  | miR-130b | Scavenger receptor class B member 1 (SCARB1) | Lipid uptake – required for entry and cell-to-cell transmission | (Singaravelu et al., 2015) |  |
|  |  | Low density lipoprotein receptor protein (LDLR) | Lipid uptake -needed for HCV replication and cell entry |  |  |
|  |  | Peroxisome proliferator-activated receptor gamma (PPARγ) | lipogenic transcription factor |  |  |
|  | miR-152 | Wnt1 (WNT1) | Proto-onco gene, promotes proliferation | (Huang et al., 2014) |  |
|  | miR-155 | T-cell immunoglobulin-3 (Tim-3) | Negative regulator of innate and adaptive immune responses | (Cheng et al., 2015b) |  |
|  | miR-181a | Dual specific phosphatase 6 (DUSP6) | Inactivator of T-cell receptor signalling | (Li et al., 2015a) |  |
|  | miR-185 | 1-acyl-sn-glycerol-3-phosphate acyltransferase gamma (AGPAT3) | key enzyme catalyzing intermediate steps in the synthesis of triglycerides from glycerol-3-phosphate | (Singaravelu et al., 2015) |  |
|  |  | Low density lipoprotein receptor protein (LDLR) | Lipid uptake -needed for HCV replication and cell entry |  |  |
|  |  | Stearoyl-CoA desaturase (SCD1) | Enzyme involved in fatty acid metabolism - required for HCV replication |  |  |
|  |  | Sterol regulatory element-binding protein 2 (SREBP2) | Involved in cholesterol biosynthesis – required for HCV replication and assembly |  |  |
|  | miR-196 | Transcription regulator protein bach1 (BACH1) | Regulate the oxidative stress pathway | (Hou et al., 2010) |  |
|  | miR-221 | Suppressor of cytokine signalling 1 (SOCS1)  Suppressor of cytokine signalling 1 (SOCS3) | Negatively regulate cytokine signalling | (Xu et al., 2014b) |  |
|  | miR-373 | Janus kinase 1 (JAK1) | Signalling molecules of the interferon response | (Mukherjee et al., 2015) |  |
|  |  | IFN-regulating factor 9 (IRF9) |  |  |  |
|  | miR-449a | Interleukin 6 receptor (IL-6R) | Receptor for the proinflammatory protein IL6 | (Sarma et al., 2014) |  |
|  | miR-758 | Toll like receptor 3 (TLR3)  Toll like receptor 7 (TLR7) | Pathogen recognition and induction of innate immune reponses | (Yang et al., 2014b) |  |
|  | miR-942 | Interferon stimulated gene 12a (ISG12a) | Involved in interferon response, negative regulator of apoptosis | (Yang et al., 2014a) |  |
| Herpes simplex 1 virus | miR-101 | mitochondrial ATP synthase subunit beta (ATP5B) | Respiration. Required for HSV replication | (Zheng et al., 2011) |  |
|  | miR-138 | Human Herpes Virus (HHV) Infected Cell Polypeptide 0 (ICP0) | viral transactivator of lytic gene expression | (Pan et al., 2014) |  |
| HIV | let-7 | Interleukin 10 (IL10) | Cytokine, moderates TNFα and IL-1α production | (Swaminathan et al., 2012) |  |
|  | miR-9 | B-lymphocyte-induced maturation protein-1 (Blimp-1) | transcriptional repressor, required for generating terminally differentiated plasma cells | (Seddiki et al., 2013) |  |
|  | miR-34a | phosphatase 1 nuclear-targeting subunit (PNUTS) | nuclear protein phosphatase 1 (PP1)-binding protein and is known to regulate cell cycle progression and apoptosis. Inhbitis HIV transcription | (Kapoor et al., 2015) |  |
|  |  | Sirtulin-1 (SIRT-1) | critical regulator of stress responses,. Deacetylates and inactivates HIV Tat protein to promote HIV gene expression and replication | (Zhang et al., 2012a) |  |
|  | miR-132 | Methyl-CpG binding protein 2 (MeCP2) | transcriptional regulatory protein | (Chiang et al., 2013) |  |
|  | miR-181a | Sterile alpha motif and histidine/aspartic acid domain-containing protein 1 (SAMHD1) | nuclear protein restriction factor, prohibits cellular infection with HIV | (Pilakka-Kanthikeel et al., 2015) |  |
|  | miR-217 | Sirtulin-1 (SIRT-1) | critical regulator of stress responses,. Deacetylates and inactivates HIV Tat protein to promote HIV gene expression and replication | (Zhang et al., 2012b) |  |
|  | miR-222 | Cluster of differentiation 4 (CD4) | Surface marker involved in leukocyte interactions | (Orecchini et al., 2014) |  |
|  | miR-891a | nuclear factor of kappa light polypeptide gene enhancer in B-cells inhibitor, alpha (IκBα) | inhibitor of nuclear transcription factor NF-κB | (Yao et al., 2015) |  |
|  | miR-1236 | Vpr (HIV-1)-binding protein (VprBP) | Cellular pro HIV factor | (Ma et al., 2014b) |  |
| Human Papilloma virus | miR-34a | NOD-like receptor family CARD domain containing 5 (NLRC5) | negatively regulates NF-κB signalling | (Li et al., 2016) |  |
|  | miR-122 | Human papilloma virus genes E6 |  |  |  |
|  | miR-145 | Human papilloma virus genes E1 and E2 |  | (Gunasekharan and Laimins, 2013) |  |
| Human T cell leukaemia virus | miR-28 | HTLV-1 genome |  | (Bai and Nicot, 2015) |  |
| Influenza | miR-17 | GalNAc transferase 3 (GALNT3) | Initiates mucin production to inhibit viral transmission | (Nakamura et al., 2016) |  |
|  | miR-24 | Furin (FUR) | Essential host protein for influenza entry into cell | (Loveday et al., 2015) |  |
|  | miR-29c | Bcl-2-like protein 2 (BCL2L2) | Negative regulator of apoptosis | (Guan et al., 2012) |  |
|  | miR-29c | A20 | Feedback inhibitor of the NF-κB pathway | (Zhang et al., 2014b) |  |
|  | miR-141 | transforming growth factor (TGF)-β2 | Cytokine that modulates leukocyte activity. Exact activity is dependent on a leukocyte and it’s activation status | (Lam et al., 2013) |  |
|  | miR-221 | GalNAc transferase 3 (GALNT3) | Initiates mucin production to inhibit viral transmission | (Nakamura et al., 2016) |  |
|  | miR-302c | NF-κB inducing kinase (NIK) | Activates type I IFN expression through the NF-κB pathway | (Gui et al., 2015) |  |
|  | miR-449b | histone deacetylase 1 (HDAC1) | Represses activation of the IFNβ promoter | (Buggele et al., 2013) |  |
|  | miR-485 | Retinoic acid-inducible gene I (RIG-I) | cytosolic sensor of viral RNA and induces an antiviral immune response. | (Ingle et al., 2015) |  |
|  |  | gene PB1 (a H5N1 influenza transcript) | RNA polymerase required for viral replication |  |  |
|  | miR-548 | influenza non-structural-1A binding protein | Binds to the influenza protein non-structural protein-1 to promote viral survival | (Othumpangat et al., 2013) |  |
|  | miR-4276 | cytochrome c oxidase VIC (COX6C) | regulator of the intrinsic apoptotic pathway | (Othumpangat et al., 2014) |  |
| Japanese encephalitis virus | miR-15b | ring finger protein 125 (RNF125) | Negative regulator of RIG-1 signallings | (Zhu et al., 2015) |  |
|  | miR-19b-3p | ring finger protein 11 (RNF11) | negative regulator of nuclear factor kappa B signalling | (Ashraf et al., 2016) |  |
|  | miR-33a | eukaryotic translation elongation factor 1A1 (EEF1A1) | Interacts with the JEV proteins to promote viral replication | (Chen et al., 2016) |  |
|  | miR-146a | Interleukin-1 receptor-associated kinase 1 (IRAK1) Interleukin-1 receptor-associated kinase 2 (IRAK2) TNF receptor associated factor (TRAF6) | Promote antiviral interferon responses | (Sharma et al., 2015) |  |
|  | miR-155 | Complement factor H (CFH) | Complement activation against pathogens | (Pareek et al., 2014) |  |
| Kaposi’s sarcoma-associated virus | miR-132 | p300 transcriptional co-activator | A transcriptional coactivator involved in the IFN-γ signaling cascade | (Lagos et al., 2010) |  |
|  | miR-146a | Chemokine receptor 4 (CXCR4) | Involved in chemotaxis | (Punj et al., 2010) |  |
|  | miR-320d | KSHV replication and transcription activator | Required for replication | (Yan et al., 2013) |  |
|  | miR-498 | KSHV replication and transcription activator | Required for replication | (Yan et al., 2013) |  |
| *Klebsiella pneumoniae* | miR-23a | High mobility group nucleosomal-binding domain 2 (HMGN2) | Regulates integrin expression and K. pneumoniae adhesion | (Teng et al., 2016) |  |
|  | miR-155 |  |  |  |  |
| *Mycobacterial spp.* | let-7f | A20 | Feedback inhibitor of the NF-κB pathway | (Kumar et al., 2015) |  |
|  | miR-26a | Histone acetyltransferase p300 (p300) | A transcriptional coactivator involved in the IFN-γ signaling cascade | (Ni et al., 2014) |  |
|  | miR-124 | Toll-like receptor 6 TLR6 | Toll like receptor signalling | (Ma et al., 2014a) |  |
|  |  | myeloid differentiation factor 88 (MyD88) |  |  |  |
|  |  | TNF receptor associated factor 6 (TRAF6) |  |  |  |
|  | miR-132 | Histone acetyltransferase p300 (p300) | A transcriptional coactivator involved in the IFN-γ signaling cascade | (Ni et al., 2014) |  |
|  | miR-146a | Interleukin-1 receptor-associated kinase 1 (IRAK1)  TNF receptor associated factor 6 (TRAF6) | Mediate TLR signalling | (Liu et al., 2014) |  |
|  |  | Prostaglandin-endoperoxidase synthase 2 (PTGS2) | key enzyme catalyzing the  rate-limiting step in the inducible production of prostaglandin E2 |  |  |
|  | miR-149 | Myeloid differentiation primary response gene 88 (MYD88) | Mediates toll like receptor signalling | (Xu et al., 2014c) |  |
|  | MiR-155 | SH2 domain-containing inositol 5-phosphatase 1 (SHIP1) | modulates leukocyte activation, proliferation and survival | (Rothchild et al., 2016) |  |
|  | miR-223 | Chemokine (C-X-C motif) ligand 2 (CXCL2) | a chemokine  involved in polymorph chemotaxis | (Dorhoi et al., 2013) |  |
|  |  | Chemokine (C-C motif) ligand 3 (CCL3) | chemokine |  |  |
|  |  | Interleukin 6 (IL-6) | Proinflammatory cytokine |  |  |
|  | miR-223 | forkhead box O3 (FOXO3) | forkhead transcription factor – promotes apoptosis | (Xi et al., 2015) |  |
| *Neisseria gonorrhoea* | miR-718 | Phosphatase and tensin homolog (PTEN) | negative regulator of the PI3K/Akt pathway which limiting Th1 polarization | (Kalantari et al., 2017) |  |
| *Plasmodium falciparum* | miR-451 | Plasmodium falciparum transcripts | Parasite survival and proiferation | (LaMonte et al., 2012) |  |
| Polio Virus | miR-555 | heterogeneous nuclear ribonucleoprotein C1/C2 (hnRNP C) | Pre-mRNA processing, mRNA metabolism and transport. Required for polio replication | (Shim et al., 2016) |  |
| Respiratory syncytial virus | miR-24 | polo-like kinase 3 (PLK3) | Cell proliferation | (Bakre et al., 2015) |  |
|  |  | casein kinase γ1 (CSNK1G1) | Cell proliferation |  |  |
|  |  | Kruppel-like factor 6 (KLF6) | transcription factor, promotes TGF-β expression |  |  |
| *Salmonella spp.* | let-7 | Interleukin 6 (IL6) | Pro-inflammatory cytokine | (Schulte et al., 2011) |  |
|  |  | Interleukin 10 (IL10) | Cytokine, moderates TNFα and IL-1α production |  |  |
|  | miR-15b | Cylin D-2 (CCND2) | Cell cycle regulator | (Maudet et al., 2014) |  |
|  | miR-29a | Caveolin 2 (CAV2) | Constituent of caveola which are lipid structures found in the cytosol and vesicles which influence bacterial uptake | (Hoeke et al., 2013) |  |
|  | miR-128 | Macrophage colony stimulating factor (MCSF) | Hematopoietic growth factor | (Zhang et al., 2014a) |  |
|  | miR-146a | Interleukin-1 receptor-associated kinase 1 (IRAK1) TNF receptor associated factor (TRAF6) | Mediate TLR signalling | (Quinn et al., 2013) |  |
| *Staphylococcus aurerus* | miR-24 | chitinase 3-like 1 (CHI3L1) | Secretory glycoprotein mediating multiple signalling pathways, upregulated in inflammation | (Jin et al., 2015) |  |
| *Toxoplasma gondii* | miR-17-92 cluster | Bcl-2-like protein 11 (BCL2L11) | Effector of apoptosis | (Cai et al., 2014) |  |
| West Nile virus | miR-532 | SEC14 and spectrin domains 1 (SESTD1) | Activates certain cation ion channels, required for calcium transport which is essential for propagation of West Nile virus | (Slonchak et al., 2016) |  |
|  |  | Transforming growth factor beta  activated kinase binding protein 3 (TAB3) | Involved in IL-1 and TNF-alpha signalling pathways |  |  |

**References for Supplementary Material Table 1**

Ashraf, U., Zhu, B., Ye, J., Wan, S., Nie, Y., Chen, Z., et al. (2016). MicroRNA-19b-3p Modulates Japanese Encephalitis Virus-Mediated Inflammation via Targeting RNF11. *J. Virol.* 90, 4780–95. doi:10.1128/JVI.02586-15.

Bai, X. T., and Nicot, C. (2015). miR-28-3p is a cellular restriction factor that inhibits human T cell leukemia virus, type 1 (HTLV-1) replication and virus infection. *J. Biol. Chem.* 290, 5381–90. doi:10.1074/jbc.M114.626325.

Bakre, A., Wu, W., Hiscox, J., Spann, K., Teng, M. N., and Tripp, R. A. (2015). Human respiratory syncytial virus non-structural protein NS1 modifies miR-24 expression via transforming growth factor-β. *J. Gen. Virol.* 96, 3179–91. doi:10.1099/jgv.0.000261.

Bandyopadhyay, S., Long, M. E., and Allen, L.-A. H. (2014). Differential expression of microRNAs in Francisella tularensis-infected human macrophages: miR-155-dependent downregulation of MyD88 inhibits the inflammatory response. *PLoS One* 9, e109525. doi:10.1371/journal.pone.0109525.

Buggele, W. A., Krause, K. E., and Horvath, C. M. (2013). Small RNA profiling of influenza A virus-infected cells identifies miR-449b as a regulator of histone deacetylase 1 and interferon beta. *PLoS One* 8, e76560. doi:10.1371/journal.pone.0076560.

Cai, Y., Chen, H., Mo, X., Tang, Y., Xu, X., Zhang, A., et al. (2014). Toxoplasma gondii inhibits apoptosis via a novel STAT3-miR-17-92-Bim pathway in macrophages. *Cell. Signal.* 26, 1204–12. doi:10.1016/j.cellsig.2014.02.013.

Castillo, J. A., Castrillón, J. C., Diosa-Toro, M., Betancur, J. G., St Laurent, G., Smit, J. M., et al. (2016). Complex interaction between dengue virus replication and expression of miRNA-133a. *BMC Infect. Dis.* 16, 29. doi:10.1186/s12879-016-1364-y.

Chen, R.-F., Yang, K. D., Lee, I.-K., Liu, J.-W., Huang, C.-H., Lin, C.-Y., et al. (2014). Augmented miR-150 expression associated with depressed SOCS1 expression involved in dengue haemorrhagic fever. *J. Infect.* 69, 366–74. doi:10.1016/j.jinf.2014.05.013.

Chen, X.-M., Splinter, P. L., O’Hara, S. P., and LaRusso, N. F. (2007). A cellular micro-RNA, let-7i, regulates Toll-like receptor 4 expression and contributes to cholangiocyte immune responses against Cryptosporidium parvum infection. *J. Biol. Chem.* 282, 28929–38. doi:10.1074/jbc.M702633200.

Chen, Y., Chen, J., Wang, H., Shi, J., Wu, K., Liu, S., et al. (2013). HCV-induced miR-21 contributes to evasion of host immune system by targeting MyD88 and IRAK1. *PLoS Pathog.* 9, e1003248. doi:10.1371/journal.ppat.1003248.

Chen, Z.-G., Liu, H., Zhang, J.-B., Zhang, S.-L., Zhao, L.-H., and Liang, W.-Q. (2015). Upregulated microRNA-214 enhances cardiac injury by targeting ITCH during coxsackievirus infection. *Mol. Med. Rep.* 12, 1258–64. doi:10.3892/mmr.2015.3539.

Chen, Z., Ye, J., Ashraf, U., Li, Y., Wei, S., Wan, S., et al. (2016). MicroRNA-33a-5p Modulates Japanese Encephalitis Virus Replication by Targeting Eukaryotic Translation Elongation Factor 1A1. *J. Virol.* 90, 3722–34. doi:10.1128/JVI.03242-15.

Cheng, J.-C., Yeh, Y.-J., Tseng, C.-P., Hsu, S.-D., Chang, Y.-L., Sakamoto, N., et al. (2012). Let-7b is a novel regulator of hepatitis C virus replication. *Cell. Mol. Life Sci.* 69, 2621–33. doi:10.1007/s00018-012-0940-6.

Cheng, M., Si, Y., Niu, Y., Liu, X., Li, X., Zhao, J., et al. (2013). High-throughput profiling of alpha interferon- and interleukin-28B-regulated microRNAs and identification of let-7s with anti-hepatitis C virus activity by targeting IGF2BP1. *J. Virol.* 87, 9707–18. doi:10.1128/JVI.00802-13.

Cheng, S. F., Li, L., and Wang, L. M. (2015a). miR-155 and miR-146b negatively regulates IL6 in Helicobacter pylori (cagA+) infected gastroduodenal ulcer. *Eur. Rev. Med. Pharmacol. Sci.* 19, 607–13.

Cheng, Y. Q., Ren, J. P., Zhao, J., Wang, J. M., Zhou, Y., Li, G. Y., et al. (2015b). MicroRNA-155 regulates interferon-γ production in natural killer cells via Tim-3 signalling in chronic hepatitis C virus infection. *Immunology* 145, 485–97. doi:10.1111/imm.12463.

Chiang, K., Liu, H., and Rice, A. P. (2013). miR-132 enhances HIV-1 replication. *Virology* 438, 1–4. doi:10.1016/j.virol.2012.12.016.

Corsten, M., Heggermont, W., Papageorgiou, A.-P., Deckx, S., Tijsma, A., Verhesen, W., et al. (2015). The microRNA-221/-222 cluster balances the antiviral and inflammatory response in viral myocarditis. *Eur. Heart J.* 36, 2909–19. doi:10.1093/eurheartj/ehv321.

Dai, X., Zhang, W., Zhang, H., Sun, S., Yu, H., Guo, Y., et al. (2014). Modulation of HBV replication by microRNA-15b through targeting hepatocyte nuclear factor 1α. *Nucleic Acids Res.* 42, 6578–90. doi:10.1093/nar/gku260.

Dorhoi, A., Iannaccone, M., Farinacci, M., Faé, K. C., Schreiber, J., Moura-Alves, P., et al. (2013). MicroRNA-223 controls susceptibility to tuberculosis by regulating lung neutrophil recruitment. *J. Clin. Invest.* 123, 4836–48. doi:10.1172/JCI67604.

Du, X., Wang, H., Xu, F., Huang, Y., Liu, Z., and Liu, T. (2015). Enterovirus 71 induces apoptosis of SH‑SY5Y human neuroblastoma cells through stimulation of endogenous microRNA let-7b expression. *Mol. Med. Rep.* 12, 953–9. doi:10.3892/mmr.2015.3482.

Escalera-Cueto, M., Medina-Martínez, I., del Angel, R. M., Berumen-Campos, J., Gutiérrez-Escolano, A. L., and Yocupicio-Monroy, M. (2015). Let-7c overexpression inhibits dengue virus replication in human hepatoma Huh-7 cells. *Virus Res.* 196, 105–12. doi:10.1016/j.virusres.2014.11.010.

Fassi Fehri, L., Koch, M., Belogolova, E., Khalil, H., Bolz, C., Kalali, B., et al. (2010). Helicobacter pylori induces miR-155 in T cells in a cAMP-Foxp3-dependent manner. *PLoS One* 5, e9500. doi:10.1371/journal.pone.0009500.

Fu, Y.-R., Liu, X.-J., Li, X.-J., Shen, Z., Yang, B., Wu, C.-C., et al. (2015). MicroRNA miR-21 attenuates human cytomegalovirus replication in neural cells by targeting Cdc25a. *J. Virol.* 89, 1070–82. doi:10.1128/JVI.01740-14.

Fukuhara, T., Kambara, H., Shiokawa, M., Ono, C., Katoh, H., Morita, E., et al. (2012). Expression of microRNA miR-122 facilitates an efficient replication in nonhepatic cells upon infection with hepatitis C virus. *J. Virol.* 86, 7918–33. doi:10.1128/JVI.00567-12.

Gao, D., Zhai, A., Qian, J., Li, A., Li, Y., Song, W., et al. (2015). Down-regulation of suppressor of cytokine signaling 3 by miR-122 enhances interferon-mediated suppression of hepatitis B virus. *Antiviral Res.* 118, 20–8. doi:10.1016/j.antiviral.2015.03.001.

Gelley, F., Zadori, G., Nemes, B., Fassan, M., Lendvai, G., Sarvary, E., et al. (2014). MicroRNA profile before and after antiviral therapy in liver transplant recipients for hepatitis C virus cirrhosis. *J. Gastroenterol. Hepatol.* 29, 121–7. doi:10.1111/jgh.12362.

Gong, A.-Y., Hu, G., Zhou, R., Liu, J., Feng, Y., Soukup, G. A., et al. (2011). MicroRNA-221 controls expression of intercellular adhesion molecule-1 in epithelial cells in response to Cryptosporidium parvum infection. *Int. J. Parasitol.* 41, 397–403. doi:10.1016/j.ijpara.2010.11.011.

Guan, Z., Shi, N., Song, Y., Zhang, X., Zhang, M., and Duan, M. (2012). Induction of the cellular microRNA-29c by influenza virus contributes to virus-mediated apoptosis through repression of antiapoptotic factors BCL2L2. *Biochem. Biophys. Res. Commun.* 425, 662–7. doi:10.1016/j.bbrc.2012.07.114.

Gui, S., Chen, X., Zhang, M., Zhao, F., Wan, Y., Wang, L., et al. (2015). Mir-302c mediates influenza A virus-induced IFNβ expression by targeting NF-κB inducing kinase. *FEBS Lett.* 589, 4112–8. doi:10.1016/j.febslet.2015.11.011.

Gunasekharan, V., and Laimins, L. A. (2013). Human papillomaviruses modulate microRNA 145 expression to directly control genome amplification. *J. Virol.* 87, 6037–43. doi:10.1128/JVI.00153-13.

Guo, H., Liu, H., Mitchelson, K., Rao, H., Luo, M., Xie, L., et al. (2011). MicroRNAs-372/373 promote the expression of hepatitis B virus through the targeting of nuclear factor I/B. *Hepatology* 54, 808–19. doi:10.1002/hep.24441.

Hao, J., Jin, W., Li, X., Wang, S., Zhang, X., Fan, H., et al. (2013). Inhibition of alpha interferon (IFN-α)-induced microRNA-122 negatively affects the anti-hepatitis B virus efficiency of IFN-α. *J. Virol.* 87, 137–47. doi:10.1128/JVI.01710-12.

Ho, B.-C. C., Yu, I.-S., Lu, L.-F., Rudensky, A., Chen, H.-Y., Tsai, C.-W., et al. (2014). Inhibition of miR-146a prevents enterovirus-induced death by restoring the production of type I interferon. *Nat. Commun.* 5, 3344. doi:10.1038/ncomms4344.

Ho, B.-C., Yu, S.-L., Chen, J. J. W., Chang, S.-Y., Yan, B.-S., Hong, Q.-S., et al. (2011). Enterovirus-induced miR-141 contributes to shutoff of host protein translation by targeting the translation initiation factor eIF4E. *Cell Host Microbe* 9, 58–69. doi:10.1016/j.chom.2010.12.001.

Hoeke, L., Sharbati, J., Pawar, K., Keller, A., Einspanier, R., and Sharbati, S. (2013). Intestinal Salmonella typhimurium infection leads to miR-29a induced caveolin 2 regulation. *PLoS One* 8, e67300. doi:10.1371/journal.pone.0067300.

Hou, W., Tian, Q., Zheng, J., and Bonkovsky, H. L. (2010). MicroRNA-196 represses Bach1 protein and hepatitis C virus gene expression in human hepatoma cells expressing hepatitis C viral proteins. *Hepatology* 51, 1494–504. doi:10.1002/hep.23401.

Hou, Z. H., Han, Q. J., Zhang, C., Tian, Z. G., and Zhang, J. (2014). miR146a impairs the IFN-induced anti-HBV immune response by downregulating STAT1 in hepatocytes. *Liver Int.* 34, 58–68. doi:10.1111/liv.12244.

Hu, G., Zhou, R., Liu, J., Gong, A.-Y., Eischeid, A. N., Dittman, J. W., et al. (2009). MicroRNA-98 and let-7 confer cholangiocyte expression of cytokine-inducible Src homology 2-containing protein in response to microbial challenge. *J. Immunol.* 183, 1617–24. doi:10.4049/jimmunol.0804362.

Huang, J.-Y., Chou, S.-F., Lee, J.-W., Chen, H.-L., Chen, C.-M., Tao, M.-H., et al. (2015). MicroRNA-130a can inhibit hepatitis B virus replication via targeting PGC1α and PPARγ. *RNA* 21, 385–400. doi:10.1261/rna.048744.114.

Huang, S., Xie, Y., Yang, P., Chen, P., and Zhang, L. (2014). HCV core protein-induced down-regulation of microRNA-152 promoted aberrant proliferation by regulating Wnt1 in HepG2 cells. *PLoS One* 9, e81730. doi:10.1371/journal.pone.0081730.

Ingle, H., Kumar, S., Raut, A. A., Mishra, A., Kulkarni, D. D., Kameyama, T., et al. (2015). The microRNA miR-485 targets host and influenza virus transcripts to regulate antiviral immunity and restrict viral replication. *Sci. Signal.* 8, ra126. doi:10.1126/scisignal.aab3183.

Jabłonowska, E., Wójcik, K., Szymańska, B., Omulecka, A., Cwiklińska, H., and Piekarska, A. (2014). Hepatic HMOX1 expression positively correlates with Bach-1 and miR-122 in patients with HCV mono and HIV/HCV coinfection. *PLoS One* 9, e95564. doi:10.1371/journal.pone.0095564.

Jangra, R. K., Yi, M., and Lemon, S. M. (2010). Regulation of hepatitis C virus translation and infectious virus production by the microRNA miR-122. *J. Virol.* 84, 6615–25. doi:10.1128/JVI.00417-10.

Jin, T., Lu, Y., He, Q. X., Wang, H., Li, B. F., Zhu, L. Y., et al. (2015). The Role of MicroRNA, miR-24, and Its Target CHI3L1 in Osteomyelitis Caused by Staphylococcus aureus. *J. Cell. Biochem.* 116, 2804–13. doi:10.1002/jcb.25225.

Jopling, C. L., Yi, M., Lancaster, A. M., Lemon, S. M., and Sarnow, P. (2005). Modulation of hepatitis C virus RNA abundance by a liver-specific MicroRNA. *Science* 309, 1577–81. doi:10.1126/science.1113329.

Kalantari, P., Harandi, O. F., Agarwal, S., Rus, F., Kurt-Jones, E. A., Fitzgerald, K. A., et al. (2017). miR-718 represses proinflammatory cytokine production through targeting phosphatase and tensin homolog (PTEN). *J. Biol. Chem.* 292, 5634–5644. doi:10.1074/jbc.M116.749325.

Kapoor, R., Arora, S., Ponia, S. S., Kumar, B., Maddika, S., and Banerjea, A. C. (2015). The miRNA miR-34a enhances HIV-1 replication by targeting PNUTS/PPP1R10, which negatively regulates HIV-1 transcriptional complex formation. *Biochem. J.* 470, 293–302. doi:10.1042/BJ20150700.

Khlaiphuengsin, A., T-Thienprasert, N. P., Tangkijvanich, P., Posuwan, N., Makkoch, J., Poovorawan, Y., et al. (2015). Human miR-5193 Triggers Gene Silencing in Multiple Genotypes of Hepatitis B Virus. *MicroRNA (Shāriqah, United Arab Emirates)* 4, 123–30.

Kohno, T., Tsuge, M., Murakami, E., Hiraga, N., Abe, H., Miki, D., et al. (2014). Human microRNA hsa-miR-1231 suppresses hepatitis B virus replication by targeting core mRNA. *J. Viral Hepat.* 21, e89-97. doi:10.1111/jvh.12240.

Kumar, M., Sahu, S. K., Kumar, R., Subuddhi, A., Maji, R. K., Jana, K., et al. (2015). MicroRNA let-7 modulates the immune response to Mycobacterium tuberculosis infection via control of A20, an inhibitor of the NF-κB pathway. *Cell Host Microbe* 17, 345–56. doi:10.1016/j.chom.2015.01.007.

Lagos, D., Pollara, G., Henderson, S., Gratrix, F., Fabani, M., Milne, R. S. B., et al. (2010). miR-132 regulates antiviral innate immunity through suppression of the p300 transcriptional co-activator. *Nat. Cell Biol.* 12, 513–9. doi:10.1038/ncb2054.

Lam, W.-Y., Yeung, A. C.-M., Ngai, K. L.-K., Li, M.-S., To, K.-F., Tsui, S. K.-W., et al. (2013). Effect of avian influenza A H5N1 infection on the expression of microRNA-141 in human respiratory epithelial cells. *BMC Microbiol.* 13, 104. doi:10.1186/1471-2180-13-104.

Lam, W. Y., Cheung, A. C. Y., Tung, C. K. C., Yeung, A. C. M., Ngai, K. L. K., Lui, V. W. Y., et al. (2015). miR-466 is putative negative regulator of Coxsackie virus and Adenovirus Receptor. *FEBS Lett.* 589, 246–54. doi:10.1016/j.febslet.2014.12.006.

LaMonte, G., Philip, N., Reardon, J., Lacsina, J. R., Majoros, W., Chapman, L., et al. (2012). Translocation of sickle cell erythrocyte microRNAs into Plasmodium falciparum inhibits parasite translation and contributes to malaria resistance. *Cell Host Microbe* 12, 187–99. doi:10.1016/j.chom.2012.06.007.

Li, A., Song, W., Qian, J., Li, Y., He, J., Zhang, Q., et al. (2013a). MiR-122 modulates type I interferon expression through blocking suppressor of cytokine signaling 1. *Int. J. Biochem. Cell Biol.* 45, 858–65. doi:10.1016/j.biocel.2013.01.008.

Li, G. Y., Zhou, Y., Ying, R. S., Shi, L., Cheng, Y. Q., Ren, J. P., et al. (2015a). Hepatitis C virus-induced reduction in miR-181a impairs CD4(+) T-cell responses through overexpression of DUSP6. *Hepatology* 61, 1163–73. doi:10.1002/hep.27634.

Li, J.-F., Dai, X.-P., Zhang, W., Sun, S.-H., Zeng, Y., Zhao, G.-Y., et al. (2015b). Upregulation of microRNA-146a by hepatitis B virus X protein contributes to hepatitis development by downregulating complement factor H. *MBio* 6. doi:10.1128/mBio.02459-14.

Li, J., Yu, L., Shen, Z., Li, Y., Chen, B., Wei, W., et al. (2016). miR-34a and its novel target, NLRC5, are associated with HPV16 persistence. *Infect. Genet. Evol.* 44, 293–9. doi:10.1016/j.meegid.2016.07.013.

Li, Q., Fang, Y., Zhu, P., Ren, C.-Y., Chen, H., Gu, J., et al. (2015c). Burkholderia pseudomallei survival in lung epithelial cells benefits from miRNA-mediated suppression of ATG10. *Autophagy* 11, 1293–307. doi:10.1080/15548627.2015.1058474.

Li, S., Xing, X., Yang, Q., Xu, H., He, J., Chen, Z., et al. (2013b). The effects of hepatitis C virus core protein on the expression of miR-122 in vitro. *Virol. J.* 10, 98. doi:10.1186/1743-422X-10-98.

Li, Y.-P., Gottwein, J. M., Scheel, T. K., Jensen, T. B., and Bukh, J. (2011). MicroRNA-122 antagonism against hepatitis C virus genotypes 1-6 and reduced efficacy by host RNA insertion or mutations in the HCV 5’ UTR. *Proc. Natl. Acad. Sci. U. S. A.* 108, 4991–6. doi:10.1073/pnas.1016606108.

Li, Y., Xie, J., Xu, X., Wang, J., Ao, F., Wan, Y., et al. (2013c). MicroRNA-548 down-regulates host antiviral response via direct targeting of IFN-λ1. *Protein Cell* 4, 130–41. doi:10.1007/s13238-012-2081-y.

Liu, Z., Zhou, G., Deng, X., Yu, Q., Hu, Y., Sun, H., et al. (2014). Analysis of miRNA expression profiling in human macrophages responding to Mycobacterium infection: induction of the immune regulator miR-146a. *J. Infect.* 68, 553–61. doi:10.1016/j.jinf.2013.12.017.

Loveday, E.-K., Diederich, S., Pasick, J., and Jean, F. (2015). Human microRNA-24 modulates highly pathogenic avian-origin H5N1 influenza A virus infection in A549 cells by targeting secretory pathway furin. *J. Gen. Virol.* 96, 30–9. doi:10.1099/vir.0.068585-0.

Luna, J. M., Scheel, T. K. H., Danino, T., Shaw, K. S., Mele, A., Fak, J. J., et al. (2015). Hepatitis C virus RNA functionally sequesters miR-122. *Cell* 160, 1099–110. doi:10.1016/j.cell.2015.02.025.

Ma, C., Li, Y., Zeng, J., Wu, X., Liu, X., and Wang, Y. (2014a). Mycobacterium bovis BCG triggered MyD88 induces miR-124 feedback negatively regulates immune response in alveolar epithelial cells. *PLoS One* 9, e92419. doi:10.1371/journal.pone.0092419.

Ma, L., Shen, C.-J., Cohen, É. A., Xiong, S.-D., and Wang, J.-H. (2014b). miRNA-1236 inhibits HIV-1 infection of monocytes by repressing translation of cellular factor VprBP. *PLoS One* 9, e99535. doi:10.1371/journal.pone.0099535.

Mansouri, S., Pan, Q., Blencowe, B. J., Claycomb, J. M., and Frappier, L. (2014). Epstein-Barr virus EBNA1 protein regulates viral latency through effects on let-7 microRNA and dicer. *J. Virol.* 88, 11166–77. doi:10.1128/JVI.01785-14.

Maudet, C., Mano, M., Sunkavalli, U., Sharan, M., Giacca, M., Förstner, K. U., et al. (2014). Functional high-throughput screening identifies the miR-15 microRNA family as cellular restriction factors for Salmonella infection. *Nat. Commun.* 5, 4718. doi:10.1038/ncomms5718.

Mosca, N., Castiello, F., Coppola, N., Trotta, M. C., Sagnelli, C., Pisaturo, M., et al. (2014). Functional interplay between hepatitis B virus X protein and human miR-125a in HBV infection. *Biochem. Biophys. Res. Commun.* 449, 141–5. doi:10.1016/j.bbrc.2014.05.009.

Mukherjee, A., Di Bisceglie, A. M., and Ray, R. B. (2015). Hepatitis C virus-mediated enhancement of microRNA miR-373 impairs the JAK/STAT signaling pathway. *J. Virol.* 89, 3356–65. doi:10.1128/JVI.03085-14.

Nachmani, D., Zimmermann, A., Oiknine Djian, E., Weisblum, Y., Livneh, Y., Khanh Le, V. T., et al. (2014). MicroRNA editing facilitates immune elimination of HCMV infected cells. *PLoS Pathog.* 10, e1003963. doi:10.1371/journal.ppat.1003963.

Nakamura, S., Horie, M., Daidoji, T., Honda, T., Yasugi, M., Kuno, A., et al. (2016). Influenza A Virus-Induced Expression of a GalNAc Transferase, GALNT3, via MicroRNAs Is Required for Enhanced Viral Replication. *J. Virol.* 90, 1788–801. doi:10.1128/JVI.02246-15.

Ni, B., Rajaram, M. V. S., Lafuse, W. P., Landes, M. B., and Schlesinger, L. S. (2014). Mycobacterium tuberculosis decreases human macrophage IFN-γ responsiveness through miR-132 and miR-26a. *J. Immunol.* 193, 4537–47. doi:10.4049/jimmunol.1400124.

Orecchini, E., Doria, M., Michienzi, A., Giuliani, E., Vassena, L., Ciafrè, S. A., et al. (2014). The HIV-1 Tat protein modulates CD4 expression in human T cells through the induction of miR-222. *RNA Biol.* 11, 334–8. doi:10.4161/rna.28372.

Othumpangat, S., Noti, J. D., and Beezhold, D. H. (2014). Lung epithelial cells resist influenza A infection by inducing the expression of cytochrome c oxidase VIc which is modulated by miRNA 4276. *Virology* 468–470, 256–64. doi:10.1016/j.virol.2014.08.007.

Othumpangat, S., Noti, J. D., Blachere, F. M., and Beezhold, D. H. (2013). Expression of non-structural-1A binding protein in lung epithelial cells is modulated by miRNA-548an on exposure to influenza A virus. *Virology* 447, 84–94. doi:10.1016/j.virol.2013.08.031.

Pan, D., Flores, O., Umbach, J. L., Pesola, J. M., Bentley, P., Rosato, P. C., et al. (2014). A neuron-specific host microRNA targets herpes simplex virus-1 ICP0 expression and promotes latency. *Cell Host Microbe* 15, 446–56. doi:10.1016/j.chom.2014.03.004.

Pareek, S., Roy, S., Kumari, B., Jain, P., Banerjee, A., and Vrati, S. (2014). MiR-155 induction in microglial cells suppresses Japanese encephalitis virus replication and negatively modulates innate immune responses. *J. Neuroinflammation* 11, 97. doi:10.1186/1742-2094-11-97.

Pilakka-Kanthikeel, S., Raymond, A., Atluri, V. S. R., Sagar, V., Saxena, S. K., Diaz, P., et al. (2015). Sterile alpha motif and histidine/aspartic acid domain-containing protein 1 (SAMHD1)-facilitated HIV restriction in astrocytes is regulated by miRNA-181a. *J. Neuroinflammation* 12, 66. doi:10.1186/s12974-015-0285-9.

Punj, V., Matta, H., Schamus, S., Tamewitz, A., Anyang, B., and Chaudhary, P. M. (2010). Kaposi’s sarcoma-associated herpesvirus-encoded viral FLICE inhibitory protein (vFLIP) K13 suppresses CXCR4 expression by upregulating miR-146a. *Oncogene* 29, 1835–44. doi:10.1038/onc.2009.460.

Quinn, E. M., Wang, J. H., O’Callaghan, G., and Redmond, H. P. (2013). MicroRNA-146a is upregulated by and negatively regulates TLR2 signaling. *PLoS One* 8, e62232. doi:10.1371/journal.pone.0062232.

Rothchild, A. C., Sissons, J. R., Shafiani, S., Plaisier, C., Min, D., Mai, D., et al. (2016). MiR-155-regulated molecular network orchestrates cell fate in the innate and adaptive immune response to Mycobacterium tuberculosis. *Proc. Natl. Acad. Sci. U. S. A.* doi:10.1073/pnas.1608255113.

Sarkar, N., Panigrahi, R., Pal, A., Biswas, A., Singh, S. P., Kar, S. K., et al. (2015). Expression of microRNA-155 correlates positively with the expression of Toll-like receptor 7 and modulates hepatitis B virus via C/EBP-β in hepatocytes. *J. Viral Hepat.* 22, 817–27. doi:10.1111/jvh.12390.

Sarma, N. J., Tiriveedhi, V., Crippin, J. S., Chapman, W. C., and Mohanakumar, T. (2014). Hepatitis C virus-induced changes in microRNA 107 (miRNA-107) and miRNA-449a modulate CCL2 by targeting the interleukin-6 receptor complex in hepatitis. *J. Virol.* 88, 3733–43. doi:10.1128/JVI.03060-13.

Schulte, L. N., Eulalio, A., Mollenkopf, H.-J., Reinhardt, R., and Vogel, J. (2011). Analysis of the host microRNA response to Salmonella uncovers the control of major cytokines by the let-7 family. *EMBO J.* 30, 1977–89. doi:10.1038/emboj.2011.94.

Seddiki, N., Phetsouphanh, C., Swaminathan, S., Xu, Y., Rao, S., Li, J., et al. (2013). The microRNA-9/B-lymphocyte-induced maturation protein-1/IL-2 axis is differentially regulated in progressive HIV infection. *Eur. J. Immunol.* 43, 510–20. doi:10.1002/eji.201242695.

Selvamani, S. P., Mishra, R., and Singh, S. K. (2014). Chikungunya virus exploits miR-146a to regulate NF-κB pathway in human synovial fibroblasts. *PLoS One* 9, e103624. doi:10.1371/journal.pone.0103624.

Shan, Y., Zheng, J., Lambrecht, R. W., and Bonkovsky, H. L. (2007). Reciprocal effects of micro-RNA-122 on expression of heme oxygenase-1 and hepatitis C virus genes in human hepatocytes. *Gastroenterology* 133, 1166–74. doi:10.1053/j.gastro.2007.08.002.

Sharma, N., Verma, R., Kumawat, K. L., Basu, A., and Singh, S. K. (2015). miR-146a suppresses cellular immune response during Japanese encephalitis virus JaOArS982 strain infection in human microglial cells. *J. Neuroinflammation* 12, 30. doi:10.1186/s12974-015-0249-0.

Shim, B.-S., Wu, W., Kyriakis, C. S., Bakre, A., Jorquera, P. A., Perwitasari, O., et al. (2016). MicroRNA-555 has potent antiviral properties against poliovirus. *J. Gen. Virol.* 97, 659–68. doi:10.1099/jgv.0.000372.

Singaravelu, R., Chen, R., Lyn, R. K., Jones, D. M., O’Hara, S., Rouleau, Y., et al. (2014). Hepatitis C virus induced up-regulation of microRNA-27: a novel mechanism for hepatic steatosis. *Hepatology* 59, 98–108. doi:10.1002/hep.26634.

Singaravelu, R., O’Hara, S., Jones, D. M., Chen, R., Taylor, N. G., Srinivasan, P., et al. (2015). MicroRNAs regulate the immunometabolic response to viral infection in the liver. *Nat. Chem. Biol.* 11, 988–93. doi:10.1038/nchembio.1940.

Skalsky, R. L., Corcoran, D. L., Gottwein, E., Frank, C. L., Kang, D., Hafner, M., et al. (2012). The viral and cellular microRNA targetome in lymphoblastoid cell lines. *PLoS Pathog.* 8, e1002484. doi:10.1371/journal.ppat.1002484.

Slonchak, A., Shannon, R. P., Pali, G., and Khromykh, A. A. (2016). Human MicroRNA miR-532-5p Exhibits Antiviral Activity against West Nile Virus via Suppression of Host Genes SESTD1 and TAB3 Required for Virus Replication. *J. Virol.* 90, 2388–402. doi:10.1128/JVI.02608-15.

Su, C., Hou, Z., Zhang, C., Tian, Z., and Zhang, J. (2011). Ectopic expression of microRNA-155 enhances innate antiviral immunity against HBV infection in human hepatoma cells. *Virol. J.* 8, 354. doi:10.1186/1743-422X-8-354.

Sullivan, R. P., Fogel, L. A., Leong, J. W., Schneider, S. E., Wong, R., Romee, R., et al. (2013). MicroRNA-155 tunes both the threshold and extent of NK cell activation via targeting of multiple signaling pathways. *J. Immunol.* 191, 5904–13. doi:10.4049/jimmunol.1301950.

Swaminathan, S., Suzuki, K., Seddiki, N., Kaplan, W., Cowley, M. J., Hood, C. L., et al. (2012). Differential regulation of the Let-7 family of microRNAs in CD4+ T cells alters IL-10 expression. *J. Immunol.* 188, 6238–46. doi:10.4049/jimmunol.1101196.

Tang, B., Xiao, B., Liu, Z., Li, N., Zhu, E.-D., Li, B.-S., et al. (2010). Identification of MyD88 as a novel target of miR-155, involved in negative regulation of Helicobacter pylori-induced inflammation. *FEBS Lett.* 584, 1481–6. doi:10.1016/j.febslet.2010.02.063.

Tang, W.-F., Huang, R.-T., Chien, K.-Y., Huang, J.-Y., Lau, K.-S., Jheng, J.-R., et al. (2016). Host MicroRNA miR-197 Plays a Negative Regulatory Role in the Enterovirus 71 Infectious Cycle by Targeting the RAN Protein. *J. Virol.* 90, 1424–38. doi:10.1128/JVI.02143-15.

Teng, G., Wang, W., Dai, Y., Wang, S., Chu, Y., and Li, J. (2013). Let-7b is involved in the inflammation and immune responses associated with Helicobacter pylori infection by targeting Toll-like receptor 4. *PLoS One* 8, e56709. doi:10.1371/journal.pone.0056709.

Teng, Y., Miao, J., Shen, X., Yang, X., Wang, X., Ren, L., et al. (2016). The modulation of MiR-155 and MiR-23a manipulates Klebsiella pneumoniae Adhesion on Human pulmonary Epithelial cells via Integrin α5β1 Signaling. *Sci. Rep.* 6, 31918. doi:10.1038/srep31918.

Tong, L., Lin, L., Wu, S., Guo, Z., Wang, T., Qin, Y., et al. (2013). MiR-10a* up-regulates coxsackievirus B3 biosynthesis by targeting the 3D-coding sequence. *Nucleic Acids Res.* 41, 3760–71. doi:10.1093/nar/gkt058.

Wang, S., Qiu, L., Yan, X., Jin, W., Wang, Y., Chen, L., et al. (2012). Loss of microRNA 122 expression in patients with hepatitis B enhances hepatitis B virus replication through cyclin G(1) -modulated P53 activity. *Hepatology* 55, 730–41. doi:10.1002/hep.24809.

Wang, S., Zhang, X., Ju, Y., Zhao, B., Yan, X., Hu, J., et al. (2013). MicroRNA-146a feedback suppresses T cell immune function by targeting Stat1 in patients with chronic hepatitis B. *J. Immunol.* 191, 293–301. doi:10.4049/jimmunol.1202100.

Wang, Y.-Q., Ren, Y.-F., Song, Y.-J., Xue, Y.-F., Zhang, X.-J., Cao, S.-T., et al. (2014). MicroRNA-581 promotes hepatitis B virus surface antigen expression by targeting Dicer and EDEM1. *Carcinogenesis* 35, 2127–33. doi:10.1093/carcin/bgu128.

Wen, B., Dai, H., Yang, Y., Zhuang, Y., and Sheng, R. (2013). MicroRNA-23b inhibits enterovirus 71 replication through downregulation of EV71 VPl protein. *Intervirology* 56, 195–200. doi:10.1159/000348504.

Wu, N., Gao, N., Fan, D., Wei, J., Zhang, J., and An, J. (2014). miR-223 inhibits dengue virus replication by negatively regulating the microtubule-destabilizing protein STMN1 in EAhy926 cells. *Microbes Infect.* 16, 911–22. doi:10.1016/j.micinf.2014.08.011.

Wu, S., He, L., Li, Y., Wang, T., Feng, L., Jiang, L., et al. (2013). miR-146a facilitates replication of dengue virus by dampening interferon induction by targeting TRAF6. *J. Infect.* 67, 329–41. doi:10.1016/j.jinf.2013.05.003.

Xi, X., Zhang, C., Han, W., Zhao, H., Zhang, H., and Jiao, J. (2015). MicroRNA-223 Is Upregulated in Active Tuberculosis Patients and Inhibits Apoptosis of Macrophages by Targeting FOXO3. *Genet. Test. Mol. Biomarkers* 19, 650–6. doi:10.1089/gtmb.2015.0090.

Xiao, B., Liu, Z., Li, B.-S., Tang, B., Li, W., Guo, G., et al. (2009). Induction of microRNA-155 during Helicobacter pylori infection and its negative regulatory role in the inflammatory response. *J. Infect. Dis.* 200, 916–25. doi:10.1086/605443.

Xing, T.-J., Xu, H.-T., Yu, W.-Q., Wang, B., and Zhang, J. (2014). MiRNA-548ah, a potential molecule associated with transition from immune tolerance to immune activation of chronic hepatitis B. *Int. J. Mol. Sci.* 15, 14411–26. doi:10.3390/ijms150814411.

Xu, C., He, X., Zheng, Z., Zhang, Z., Wei, C., Guan, K., et al. (2014a). Downregulation of microRNA miR-526a by enterovirus inhibits RIG-I-dependent innate immune response. *J. Virol.* 88, 11356–68. doi:10.1128/JVI.01400-14.

Xu, G., Yang, F., Ding, C.-L., Wang, J., Zhao, P., Wang, W., et al. (2014b). MiR-221 accentuates IFN׳s anti-HCV effect by downregulating SOCS1 and SOCS3. *Virology* 462–463, 343–50. doi:10.1016/j.virol.2014.06.024.

Xu, G., Zhang, Z., Xing, Y., Wei, J., Ge, Z., Liu, X., et al. (2014c). MicroRNA-149 negatively regulates TLR-triggered inflammatory response in macrophages by targeting MyD88. *J. Cell. Biochem.* 115, 919–27. doi:10.1002/jcb.24734.

Xu, L.-J., Jiang, T., Zhao, W., Han, J.-F., Liu, J., Deng, Y.-Q., et al. (2014d). Parallel mRNA and microRNA profiling of HEV71-infected human neuroblastoma cells reveal the up-regulation of miR-1246 in association with DLG3 repression. *PLoS One* 9, e95272. doi:10.1371/journal.pone.0095272.

Yan, Q., Li, W., Tang, Q., Yao, S., Lv, Z., Feng, N., et al. (2013). Cellular microRNAs 498 and 320d regulate herpes simplex virus 1 induction of Kaposi’s sarcoma-associated herpesvirus lytic replication by targeting RTA. *PLoS One* 8, e55832. doi:10.1371/journal.pone.0055832.

Yang, D., Meng, X., Xue, B., Liu, N., Wang, X., and Zhu, H. (2014a). MiR-942 mediates hepatitis C virus-induced apoptosis via regulation of ISG12a. *PLoS One* 9, e94501. doi:10.1371/journal.pone.0094501.

Yang, Q., Fu, S., and Wang, J. (2014b). Hepatitis C virus infection decreases the expression of Toll-like receptors 3 and 7 via upregulation of miR-758. *Arch. Virol.* 159, 2997–3003. doi:10.1007/s00705-014-2167-3.

Yao, S., Hu, M., Hao, T., Li, W., Xue, X., Xue, M., et al. (2015). MiRNA-891a-5p mediates HIV-1 Tat and KSHV Orf-K1 synergistic induction of angiogenesis by activating NF-κB signaling. *Nucleic Acids Res.* 43, 9362–78. doi:10.1093/nar/gkv988.

Ye, X., Hemida, M. G., Qiu, Y., Hanson, P. J., Zhang, H. M., and Yang, D. (2013). MiR-126 promotes coxsackievirus replication by mediating cross-talk of ERK1/2 and Wnt/β-catenin signal pathways. *Cell. Mol. Life Sci.* 70, 4631–44. doi:10.1007/s00018-013-1411-4.

Yu, H., Lu, J., Zuo, L., Yan, Q., Yu, Z., Li, X., et al. (2012). Epstein-Barr virus downregulates microRNA 203 through the oncoprotein latent membrane protein 1: a contribution to increased tumor incidence in epithelial cells. *J. Virol.* 86, 3088–99. doi:10.1128/JVI.05901-11.

Zhang, H.-S., Chen, X.-Y., Wu, T.-C., Sang, W.-W., and Ruan, Z. (2012a). MiR-34a is involved in Tat-induced HIV-1 long terminal repeat (LTR) transactivation through the SIRT1/NFκB pathway. *FEBS Lett.* 586, 4203–7. doi:10.1016/j.febslet.2012.10.023.

Zhang, H.-S., Wu, T.-C., Sang, W.-W., and Ruan, Z. (2012b). MiR-217 is involved in Tat-induced HIV-1 long terminal repeat (LTR) transactivation by down-regulation of SIRT1. *Biochim. Biophys. Acta* 1823, 1017–23. doi:10.1016/j.bbamcr.2012.02.014.

Zhang, T., Yu, J., Zhang, Y., Li, L., Chen, Y., Li, D., et al. (2014a). Salmonella enterica serovar enteritidis modulates intestinal epithelial miR-128 levels to decrease macrophage recruitment via macrophage colony-stimulating factor. *J. Infect. Dis.* 209, 2000–11. doi:10.1093/infdis/jiu006.

Zhang, X., Dong, C., Sun, X., Li, Z., Zhang, M., Guan, Z., et al. (2014b). Induction of the cellular miR-29c by influenza virus inhibits the innate immune response through protection of A20 mRNA. *Biochem. Biophys. Res. Commun.* 450, 755–61. doi:10.1016/j.bbrc.2014.06.059.

Zheng, S., Li, Y., Zhang, Y., Li, X., and Tang, H. (2011). MiR-101 regulates HSV-1 replication by targeting ATP5B. *Antiviral Res.* 89, 219–26. doi:10.1016/j.antiviral.2011.01.008.

Zheng, Z., Ke, X., Wang, M., He, S., Li, Q., Zheng, C., et al. (2013). Human microRNA hsa-miR-296-5p suppresses enterovirus 71 replication by targeting the viral genome. *J. Virol.* 87, 5645–56. doi:10.1128/JVI.02655-12.

Zhu, B., Ye, J., Nie, Y., Ashraf, U., Zohaib, A., Duan, X., et al. (2015). MicroRNA-15b Modulates Japanese Encephalitis Virus-Mediated Inflammation via Targeting RNF125. *J. Immunol.* 195, 2251–62. doi:10.4049/jimmunol.1500370.

**
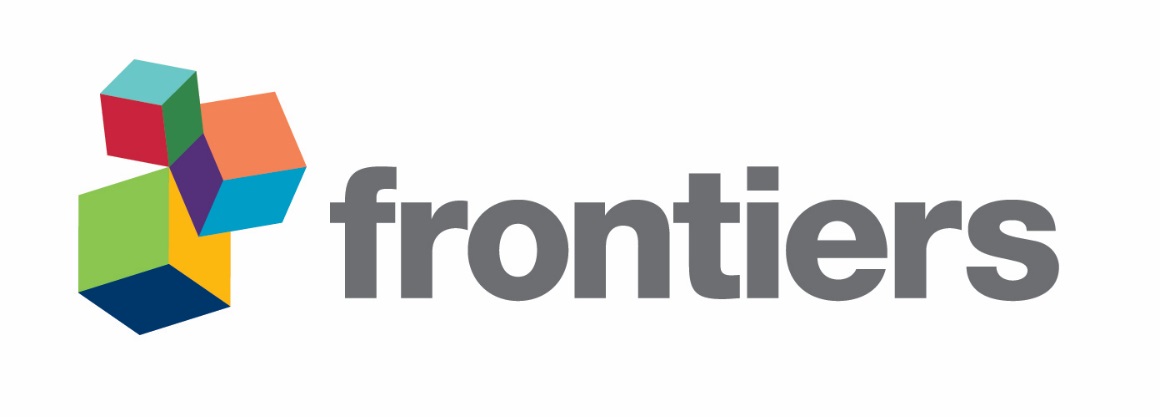
**
