# Supplementary material for: The Clinical Application of MicroRNAs in Infectious Disease
Source: Front Immunol. 2017 Sep 25;8:1182. doi: 10.3389/fimmu.2017.01182 (PMC5622146; doi:10.3389/fimmu.2017.01182)
Supplement: Supplementary file 2 [file Table_2.DOCX]

Supplementary Material: Table 2

**The Clinical Application of MicroRNA in infectious Disease.**

**Authors: Ruth Drury MBChB^1*^, Daniel O’Connor DPhil ^1^, Andrew J Pollard FMedSci^1^**

^1.^ Oxford Vaccine Group, Centre for Clinical Vaccinology and Tropical Medicine, Department of Paediatrics, University of Oxford, The Churchill Hospital, Old Road, Oxford OX3 7LE, UK

**Corresponding Author:** Ruth Drury, Oxford Vaccine Group, Centre for Clinical Vaccinology and Tropical Medicine, Department of Paediatrics, University of Oxford, The Churchill Hospital, Old Road, Oxford OX3 7LJ, UK; email: ruth.drury@paediatrics.ox.ac.uk; Tel: 01865857420.

| Supplementary Table 2.  MicroRNA profiling studies investing potential microRNA biomarkers of infectious Disease Studies were identified in PubMed from results of the overall search strategy for this review and via the references of other papers:  PubMed Search strategy = ((((microRNA[Title/Abstract]) OR miR[Title/Abstract]) OR non coding RNA[Title/Abstract])) AND ((((((infection) OR infectious disease) OR virus) OR bacteria) OR fungus) OR parasite), limited to humans, up to 14^th^ July 2017. 56 out of 57 studies were included in these tables: one study was excluded because there was no RT-PCR confirmation of the microRNA profiling results. | | | | | | | | | | |
| --- | --- | --- | --- | --- | --- | --- | --- | --- | --- | --- |
| Infection | Fluid | Cases | Controls/ comparator | Micronome platform | Reference RNA in RT PCR validation | Confirmation cohort | Up-regulated miRNA(s) | Down-regulated miRNA(s) | Notes | Authors |
| HBV | serum | Chronic HBV with liver cirrhosis  N=185 | Chronic Hep B without liver cirrhosis  N=178 | miRNA RT-PCR array | Spike in Cel-miR-67 | yes |  | 18a-5p, 21-5p, 29c-3p, 106b-5p, 122-5p, 185-5p | AUC for these 6 miRNA = 0.856, sensitivity of 85%, Specificity = 70% | (Jin et al., 2015) |
| HBV |  | Chronic HBV without liver cirrhosis  N=185 | Healthy controls  N=178 | miRNA RT-PCR array | Spike in Cel-miR-67 | yes | 21-5p, 27a-3p, 122-5p, 146a-5p |  | AUC for these 4 miRNA combined = 0.997 |  |
| HBV | serum | HBV  N=186 | Healthy control  N=22 | microarray | Spike in cel-miR-238 | yes | 122, 99a, 125b, 720, 22, 1275 |  | p-values adjusted for false discovery rate  miRNA selected for validation based array data and the literature | (Akamatsu et al., 2015) |
| HBV | Serum | HBeAg +ve  N=82 | HBeAg –ve  N=104 | microarray | Spike in cel-miR-238 | Yes | 99a, 125b, 122, 720, 22 |  |  |  |
| HCV | serum | HCV  N=107 | Healthy control  N=22 | microarray | Spike in cel-miR-238 | Yes | 122, 720 |  |  |  |
| HBV | serum | Chronic HBV  N=102 | Inactive carriers  N=39 | RT-PCR array | endogenous normalisers 126-3p, 335, 320 | yes | 122-5p,192-5p 99a-5p 148a-3p |  | Bonferoni correction applied  miR-B-Index created using 122-5p, miR-99a-5p, miR-192-5 and the endogenous normalisers 126-3p, 335, and 320 which gave 100% sensitivity and specificity 72-85% for identifying inactive carriers from chronic HBV.  Improvement in MiR-B-index score correlated with a successful response to treatment, and provided additional information beyond usual parameters of response, e.g. ALT, HBsAg titre. Increased miR-B index did not correlate with other markers of liver cell death.  miR-30e-3p upregulated during PEG-IFN treatment | (Brunetto et al., 2014) |
| HBV | serum | HBV  N=51 | Healthy control  N=12 | RT-PCR arrays | Endogenous U6 | Yes | 194, 92a, 106a, 20a, let-7b, 16, 122 |  | Evidence that miRNA expression is globally increased as symptoms continue - but could also be a batch problem as not enough detail to evaluate this potential confounder | (Ji et al., 2011) |
| HBV | Plasma | Chronic HBV e-antigen positive  N=41 | Chronic HBV e-antigen negative  N=45 | miRNA RT-PCT arrays | Endogenous miR-93-5p and miR 425-3p, selected from array data | Yes | 125b-5p 192-5p, 194-5p |  |  | (van der Ree et al., 2017) |
| HBV | Plasma | Chronic HBV e-antigen negative, treatment responders  N=41 | Chronic HBV e-antigen negative, treatment non-responders  N= | miRNA RT-PCT arrays | Endogenous miR-93-5p and miR 425-3p, selected from array data | Yes | 145-5p |  | Treatment response defined as loss of HBsAg 72 weeks after starting treatment (PEG-IFN and adefovir for 48 weeks) |  |
| HCV | serum | HCV viraemia  N=39 | Healthy controls  N=29 | miRNA RT-PCR array | Endogenous U6 | No | 122-5p, 134, 424-3p, 629-5p |  | Participant’s samples pooled prior to miRNA profiling. | (Zhang et al., 2015) |
| HCV | plasma | Intravenous drug users post contraction of acute HCV  N=50 | Paired samples: Intravenous drug users pre contraction of acute HCV  N=50 | RT-PCR array | Spike in ath-miR-159a | yes | 122, 885-5p | 494 | **Cohort study:** Injection drug users recruited pre HCV infection  RT-PCR with miR-122 and miR-885-5p showed good correlation with array RT-PCR results  Multiple testing correction applied except for miR-122, miR-885-5p, let-7b which were pre-specified | (El-Diwany et al., 2015) |
|  |  |  |  |  |  |  |  |  | Changes not related to cell lysis based on correlates of cell death. Functional work showed miR-122, miR-885 increased outside the cell but not intracellularly, and down regulation of miR-494 was associated with retention of miR-494 intracellularly |  |
| HCV | serum | HCV with varying levels of fibrosis  N=44 | Healthy controls N=22 and patients with non HCV related liver fibrosis  N=20 | RT-PCR array | Spike in cel-miR-39 &  Endogenous miR-574-3p (selected from array data) | Yes | 20a,  92a |  | miR-20a: AUC= 0.704±0.067 (95%CI=0.571–0.836) sensitivity of 61.4%, specificity of 81.8%. No correlation with viral load  miR-92a AUC= 0.787±0.058 (95%CI=0.672–0.901) sensitivity of 70.5%, specificity of 77.3% for healthy controls versus HCV infected No correlation with viral load  miR-122 and miR-21 upregulated in array but not taken on for subsequent analysis due to conflicting results in the literature and possible non-specific nature of upregulation in liver injury per se. | (Shrivastava et al., 2013) |
| HCV | serum | HCV  N=36 | Healthy controls  N=15 | Microarray | no control used, total  standardised RNA input for each sample | yes | 134, 320c and 483-5p |  | Difficult to quantify total RNA in serum, especially small RNA. | (Shwetha et al., 2013) |
| TB | Serum | Active TB  N=15 | Latent TB  N=14 | Sequencing | Spike in  C-elegan miRNAs | No | 196b, 376c |  | Pooled serum samples only  paradoxically, miR-376c was not upregulated in active TB versus healthy controls who had received a BCG | (Zhang et al., 2014) |
| TB | Serum | Pulmonary TB  N=128 | Healthy controls  N=108 | Sequencing | Endogenous  miR-16 | Yes | 378, 483-5p, 22, 29c | 101,  320b | Combined 6 microRNA signature- ability to differentiate pulmonary TB from healthy controls: AUC = 0.982, sensitivity 95%, specificity 91.8% | (Zhang et al., 2013) |
| TB | Serum | Pulmonary TB  N=128 | Non TB lung pathology (pneumonia, lung cancer, COPD)  N=90 | Sequencing | Endogenous  miR-16 | Yes | 378, 483-5p, 22, 29c | 101,  320b |  |  |
| TB | Serum | Pulmonary TB  N=75 | Healthy controls  N=52 | microarray | Endogenous U6 | No | 93*, 29a | 3125 | Pooled serum  miR-29a was also upregulated in saliva | (Fu et al., 2011) |
| TB | Serum | Pulmonary TB  N=75 | Healthy controls  N=52 | microarray | Endogenous U6 | No | 29a |  |  |  |
| TB | Serum | Pulmonary TB  N=50 | Healthy controls  N=85 | RT-PCR arrays | Endogenous  miR-16 | Yes | 361-5p, 889, 567-3p, 210, 26a, 432, 134 |  | AUC = 0.863 differentiating pulmonary TB from healthy controls | (Qi et al., 2012) |
| TB | Serum | Pulmonary TB  N=50 | Enterovirus, pertussis, varicella  N=60 | RT-PCR arrays | Endogenous  miR-16 | Yes | 361-5p, 889, 567-3p, 210, 26a, 432, 134 |  | miR-26a, miR-432, miR-134 signature gave AUC =0.7 for differentiating pulmonary TB from the enterovirus/pertussis/ varicella group |  |
| TB | Sputum | Pulmonary TB  N= 58 | Healthy controls  N=32 | Microarrays | Endogenous  U6 | No | 3179, 147 | 19b-2* |  | (Yi et al., 2012) |
| TB | PBMCs | Pulmonary TB  N=28 | Healthy controls  N=24 | Microarray | Endogenous  U6 | Yes | 29 | 1,155,31,146a,10a,125b,150 | AUC to predict pulmonary TB infection when all miRNA combined:0.996 | (Zhou et al., 2016) |
| TB | Whole blood | Latent/pulmonary TB  N=33 | Healthy controls  N=16 | Microarray | Endogenous  U6, RNU48 | Yes | 21 | 150 |  | (Latorre et al., 2015) |
| TB | Whole blood | pulmonary TB  N=17 | Latent TB  N=17 | Microarray | Endogenous  U6, RNU48 | Yes | 150 | 21,29c,194 |  |  |
| TB | Whole blood | latent TB  N=17 | Healthy control N=17 | Microarray | Endogenous  U6, RNU48 | Yes |  | 150, 21 |  |  |
| Sepsis | serum | sepsis ICU 28day non survivors at  N=97 | sepsis ICU 28 day survivors  N=117 | Solexa Sequencing | Endogenous  U6 | Yes | 16, 223 | 15a, 193b*, 122, 483-5p | AUC of miR-15a, miR-16, miR-193b*, and miR-483-5p plus SOFA and APACHE II scores = 0.953 (95%CI: 0.923–0.985). Optimal sensitivity and specificity = 88.5% and 90.4% respectively. Higher than if only combined SOFA and APACHE II scores were used (AUC 0.891 (95%CI: 0.843–0.939) | (Wang et al., 2012b) |
| sepsis | serum | Sepsis survivors  N=12 | Sepsis non survivors  N=12 | Microarray | Endogenous  5sRNA | Yes | 297 | 574-5p | Best AUC for a single microRNA was miR-574-5p: AUC 0.736 | (Wang et al., 2012a) |
| Sepsis | Serum | Sepsis  N=70 | Systemic inflammatory response  N=30 | Microarray | Endogenous  miR-16 | Yes |  | 25 | AUC for miR-25 (AUC=0.806, CI 0.86, 0.95) was higher than for CRP and procalcitonin  Decreasing miR25 levels correlated with worsening sepsis and associated with mortality at 28 days | (Yao et al., 2015) |
| influenza A H1N1 | serum | severely ill  N=8 | milder disease  N=8 | RT-PCR arrays | Endogenous  U6 | no | 150 | 22 |  | (Morán et al., 2015) |
| influenza A H1N1 | serum | H1N1 infection  N=8 | Healthy controls  N=8 | RT-PCR arrays | Endogenous  U6 | no | 29, 210, 145 |  |  |  |
| Influenza A | whole bloods | Hospitalised H1N1/H3N2  N=50 | Healthy controls  N=23 | microarray | Endogenous RNUB44 | yes | 1260  299-5p, 335*, 664 | 185*, 1285, 18a  26a, 30a, 34b, 576-3p, 628-3p  665, 765 | Bonferoni corrected p values  193/334 miRNA were significantly altered according to microarray data – indicating widespread change in miRNA expression. | Tambyah (Tambyah et al., 2013) |
| H797 | serum | H7N9 infection  N=21 | Healthy controls  N=36 | RT-PCR arrays | Spike in  Cel-miR-238 | yes | 17, 20a, 106a, 376c |  | AUC when miRNA combined = 0.96 (CI 0.917-1) | (Zhu et al., 2014) |
| Varicella (non vaccinated) | serum | Varicella  N=29 | Healthy controls  N=43 | RT-PCR arrays | Spike in  Cel-miR-238 | no | 197, 363, 629, 132, 122 |  | AUC when miRNA combined = 0.872, sensitivity= 93.1, specificity 72.1%  Gene ontology analysis performed | (Qi et al., 2014) |
| Varicella (non vaccinated) | serum | Varicella  N=29 | Children with enterovirus, pertussis, TB  N=100 | RT-PCR arrays | Spike in Cel-miR-238 | no | 363, 329 |  | P-values not given |  |
| Herpes Zoster | Serum | Herpes Zoster  N=41 | Healthy controls  N=35 | RT-PCR arrays | Spike in  Cel-miR-39 | no | 190b, 571, 1276, 1303, 943, 661, |  | AUC when expression of all 6 miRs combined = 0.939 | (Li et al., 2016) |
| Dengue | Serum | Dengue  N=45 | Healthy controls  N=35 | RT-PCR arrays | Endogenous miR-191-5p Selected from array data  Plus  miR-16 | no | 21-5p, 590-5p, 188-5p, 152-3p | 146-a | AUC miR-21 = 0.90 (95% CI: 0.8303-0.9712)  AUC miR-146a = 0.742 (95% CI: 0.8303-0.9712),  hsa-miR-21-5p negatively correlated with white cell count  hsa-miR-146a-5p positively correlated with white cell count | (Ouyang et al., 2016) |
| HIV | serum | chronically infected  N=10 | Elite controllers  N=10 | RT-PCR arrays | Endogenous U6 and 5s rRNA | No | -29b-3p, -33a-5p -146a-5p |  | FDR correction applied  Levels of miR- 29b-3p and miR-33a-5p is independent of antiretroviral treatment  miRNA in elite controllers was similar to healthy controls therefore these miRNA may reflect rather than mediate control of HIV | (Reynoso et al., 2014) |
| HIV encephalitis | CSF | HIV encephalitis  N=4 | Non viral acute disseminated encephalitis  N=10 | RT-PCR arrays | Endogenous miR-622 and miR-1266 | No | 182*, 362-5p, 720, 937 |  |  | (Pacifici et al., 2013) |
| Enterovirus-71 | serum | EV71  N=46 | Healthy controls  N=41 | RT-PCR arrays | Spike in  Cel-miR-238 | no | 148a, 628-3p, 143, 324-3p, 206, 140-5p, 455-5p and 362-3p |  | Serum pooled  AUC when Ct values of miR-148a, miR-143, miR-324-3p, miR-628-3p, miR-140-5p, and miR-362-3p were subjected to multiple logistic regression analysis=0.989 | (Cui et al., 2011) |
| pertussis | serum | Pertussis  N= 66 | Healthy controls  N=68 | RT-PCR arrays | Spike in  Cel-miR-238 | No |  | 202, 342-5p, 206, 487b, 576-5p | Risk score analysis based on five microRNAs gave an AUC of 0.98 with an optimal sensitivity of 97% and specificity of 94% | (Ge et al., 2013) |
| pertussis | serum | Pertussis  N= 66 | TB, measles, mumps, varicella, enterovirus  N= not stated | RT-PCR arrays | Spike in  Cel-miR-238 | no | 202, 342-5p, 206, 487b, 576p |  | No p-values quoted |  |
| Syphilis | serum | Syphilis  N=44 | Healthy controls  N=16 | microarray | Endogenous  miR-6510-3p  (selected from array data) | yes |  | 19b-3p, 21-5p, 16-5p |  | (Lu et al., 2017) |

**References for Supplementary Material**

Akamatsu, S., Hayes, C. N., Tsuge, M., Miki, D., Akiyama, R., Abe, H., et al. (2015). Differences in serum microRNA profiles in hepatitis B and C virus infection. *J. Infect.* 70, 273–87. doi:10.1016/j.jinf.2014.10.017.

Brunetto, M. R., Cavallone, D., Oliveri, F., Moriconi, F., Colombatto, P., Coco, B., et al. (2014). A serum microRNA signature is associated with the immune control of chronic hepatitis B virus infection. *PLoS One* 9, e110782. doi:10.1371/journal.pone.0110782.

Cui, L., Qi, Y., Li, H., Ge, Y., Zhao, K., Qi, X., et al. (2011). Serum microRNA expression profile distinguishes enterovirus 71 and coxsackievirus 16 infections in patients with hand-foot-and-mouth disease. *PLoS One* 6, e27071. doi:10.1371/journal.pone.0027071.

El-Diwany, R., Wasilewski, L. N., Witwer, K. W., Bailey, J. R., Page, K., Ray, S. C., et al. (2015). Acute Hepatitis C Virus Infection Induces Consistent Changes in Circulating MicroRNAs That Are Associated with Nonlytic Hepatocyte Release. *J. Virol.* 89, 9454–64. doi:10.1128/JVI.00955-15.

Fu, Y., Yi, Z., Wu, X., Li, J., and Xu, F. (2011). Circulating microRNAs in patients with active pulmonary tuberculosis. *J. Clin. Microbiol.* 49, 4246–51. doi:10.1128/JCM.05459-11.

Ge, Y., Zhao, K., Qi, Y., Min, X., Shi, Z., Qi, X., et al. (2013). Serum microRNA expression profile as a biomarker for the diagnosis of pertussis. *Mol. Biol. Rep.* 40, 1325–32. doi:10.1007/s11033-012-2176-9.

Ji, F., Yang, B., Peng, X., Ding, H., You, H., and Tien, P. (2011). Circulating microRNAs in hepatitis B virus-infected patients. *J. Viral Hepat.* 18, e242-51. doi:10.1111/j.1365-2893.2011.01443.x.

Jin, B.-X., Zhang, Y.-H., Jin, W.-J., Sun, X.-Y., Qiao, G.-F., Wei, Y.-Y., et al. (2015). MicroRNA panels as disease biomarkers distinguishing hepatitis B virus infection caused hepatitis and liver cirrhosis. *Sci. Rep.* 5, 15026. doi:10.1038/srep15026.

Latorre, I., Leidinger, P., Backes, C., Domínguez, J., de Souza-Galvão, M. L., Maldonado, J., et al. (2015). A novel whole-blood miRNA signature for a rapid diagnosis of pulmonary tuberculosis. *Eur. Respir. J.* 45, 1173–6. doi:10.1183/09031936.00221514.

Li, X., Huang, Y., Zhang, Y., and He, N. (2016). Evaluation of microRNA Expression in Patients with Herpes Zoster. *Viruses* 8, 326. doi:10.3390/v8120326.

Lu, P., Fang, C., Cheng, Q., Ke, W.-J., Huang, T., Zhang, J., et al. (2017). Serum microRNA profiles in patients with syphilis. *J. Eur. Acad. Dermatol. Venereol.* doi:10.1111/jdv.14116.

Morán, J., Ramírez-Martínez, G., Jiménez-Alvarez, L., Cruz, A., Pérez-Patrigeon, S., Hidalgo, A., et al. (2015). Circulating levels of miR-150 are associated with poorer outcomes of A/H1N1 infection. *Exp. Mol. Pathol.* 99, 253–61. doi:10.1016/j.yexmp.2015.07.001.

Ouyang, X., Jiang, X., Gu, D., Zhang, Y., Kong, S. K., Jiang, C., et al. (2016). Dysregulated Serum MiRNA Profile and Promising Biomarkers in Dengue-infected Patients. *Int. J. Med. Sci.* 13, 195–205. doi:10.7150/ijms.13996.

Pacifici, M., Delbue, S., Ferrante, P., Jeansonne, D., Kadri, F., Nelson, S., et al. (2013). Cerebrospinal fluid miRNA profile in HIV-encephalitis. *J. Cell. Physiol.* 228, 1070–5. doi:10.1002/jcp.24254.

Qi, Y., Cui, L. L. L. L., Ge, Y., Shi, Z., Zhao, K., Guo, X., et al. (2012). Altered serum microRNAs as biomarkers for the early diagnosis of pulmonary tuberculosis infection. *BMC Infect. Dis.* 12, 384. doi:10.1186/1471-2334-12-384.

Qi, Y., Zhu, Z., Shi, Z., Ge, Y., Zhao, K., Zhou, M., et al. (2014). Dysregulated microRNA expression in serum of non-vaccinated children with varicella. *Viruses* 6, 1823–36. doi:10.3390/v6041823.

Reynoso, R., Laufer, N., Hackl, M., Skalicky, S., Monteforte, R., Turk, G., et al. (2014). MicroRNAs differentially present in the plasma of HIV elite controllers reduce HIV infection in vitro. *Sci. Rep.* 4, 5915. doi:10.1038/srep05915.

Shrivastava, S., Petrone, J., Steele, R., Lauer, G. M., Di Bisceglie, A. M., and Ray, R. B. (2013). Up-regulation of circulating miR-20a is correlated with hepatitis C virus-mediated liver disease progression. *Hepatology* 58, 863–71. doi:10.1002/hep.26296.

Shwetha, S., Gouthamchandra, K., Chandra, M., Ravishankar, B., Khaja, M. N., and Das, S. (2013). Circulating miRNA profile in HCV infected serum: novel insight into pathogenesis. *Sci. Rep.* 3, 1555. doi:10.1038/srep01555.

Tambyah, P. A., Sepramaniam, S., Mohamed Ali, J., Chai, S. C., Swaminathan, P., Armugam, A., et al. (2013). microRNAs in circulation are altered in response to influenza A virus infection in humans. *PLoS One* 8, e76811. doi:10.1371/journal.pone.0076811.

van der Ree, M. H., Jansen, L., Kruize, Z., van Nuenen, A. C., van Dort, K. A., Takkenberg, R. B., et al. (2017). Plasma MicroRNA Levels Are Associated With Hepatitis B e Antigen Status and Treatment Response in Chronic Hepatitis B Patients. *J. Infect. Dis.* 215, 1421–1429. doi:10.1093/infdis/jix140.

Wang, H., Meng, K., Chen, W. jun, Feng, D., Jia, Y., and Xie, L. (2012a). Serum miR-574-5p: a prognostic predictor of sepsis patients. *Shock* 37, 263–7. doi:10.1097/SHK.0b013e318241baf8.

Wang, H., Zhang, P., Chen, W., Feng, D., Jia, Y., and Xie, L. (2012b). Serum microRNA signatures identified by Solexa sequencing predict sepsis patients’ mortality: a prospective observational study. *PLoS One* 7, e38885. doi:10.1371/journal.pone.0038885.

Yao, L., Liu, Z., Zhu, J., Li, B., Chai, C., and Tian, Y. (2015). Clinical evaluation of circulating microRNA-25 level change in sepsis and its potential relationship with oxidative stress. *Int. J. Clin. Exp. Pathol.* 8, 7675–84.

Yi, Z., Fu, Y., Ji, R., Li, R., and Guan, Z. (2012). Altered microRNA Signatures in Sputum of Patients with Active Pulmonary Tuberculosis. *PLoS One* 7, e43184. doi:10.1371/journal.pone.0043184.

Zhang, H., Sun, Z., Wei, W., Liu, Z., Fleming, J., Zhang, S., et al. (2014). Identification of serum microRNA biomarkers for tuberculosis using RNA-seq. *PLoS One* 9, e88909. doi:10.1371/journal.pone.0088909.

Zhang, S., Ouyang, X., Jiang, X., Gu, D., Lin, Y., Kong, S. K., et al. (2015). Dysregulated Serum MicroRNA Expression Profile and Potential Biomarkers in Hepatitis C Virus-infected Patients. *Int. J. Med. Sci.* 12, 590–8. doi:10.7150/ijms.11525.

Zhang, X., Guo, J., Fan, S., Li, Y., Wei, L., Yang, X., et al. (2013). Screening and identification of six serum microRNAs as novel potential combination biomarkers for pulmonary tuberculosis diagnosis. *PLoS One* 8, e81076. doi:10.1371/journal.pone.0081076.

Zhou, M., Yu, G., Yang, X., Zhu, C., Zhang, Z., and Zhan, X. (2016). Circulating microRNAs as biomarkers for the early diagnosis of childhood tuberculosis infection. *Mol. Med. Rep.* 13, 4620–6. doi:10.3892/mmr.2016.5097.

Zhu, Z., Qi, Y., Ge, A., Zhu, Y., Xu, K., Ji, H., et al. (2014). Comprehensive characterization of serum microRNA profile in response to the emerging avian influenza A (H7N9) virus infection in humans. *Viruses* 6, 1525–39. doi:10.3390/v6041525.

**
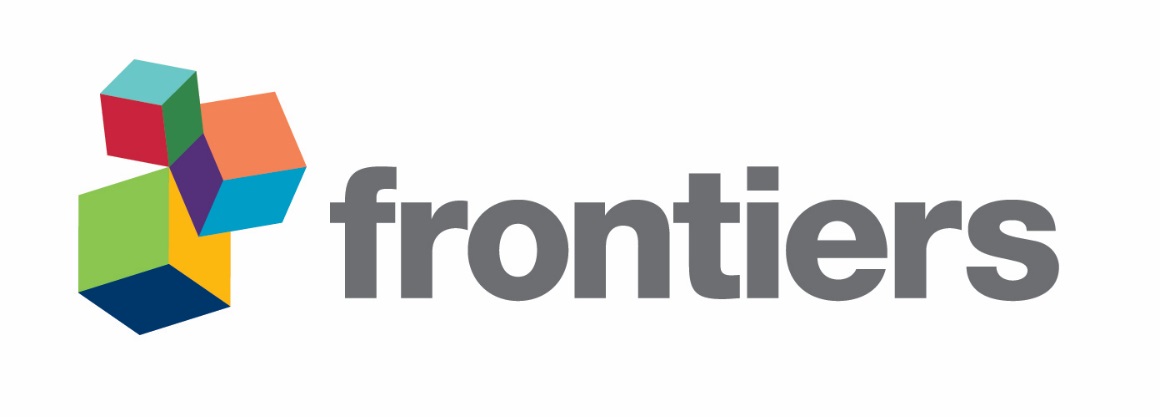
**
