# Supplementary material for: The Clinical Application of MicroRNAs in Infectious Disease
Source: Front Immunol. 2017 Sep 25;8:1182. doi: 10.3389/fimmu.2017.01182 (PMC5622146; doi:10.3389/fimmu.2017.01182)
Supplement: Supplementary file 3 [file Table_3.DOCX]

Supplementary Material

**The Clinical Application of MicroRNA in infectious Disease.**

**Authors: Ruth Drury MBChB^1*^, Daniel O’Connor DPhil ^1^, Andrew J Pollard FMedSci^1^**

^1.^ Oxford Vaccine Group, Centre for Clinical Vaccinology and Tropical Medicine, Department of Paediatrics, University of Oxford, The Churchill Hospital, Old Road, Oxford OX3 7LE, UK

**Corresponding Author:** Ruth Drury, Oxford Vaccine Group, Centre for Clinical Vaccinology and Tropical Medicine, Department of Paediatrics, University of Oxford, The Churchill Hospital, Old Road, Oxford OX3 7LJ, UK; email: ruth.drury@paediatrics.ox.ac.uk; Tel: 01865857420.

| **Supplementary Table 3: Candidate microRNA biomarker studies of infectious diseases**  Studies were identified in PubMed from results of the overall search strategy for this review and via the references of other papers:  PubMed Search strategy = ((((microRNA[Title/Abstract]) OR miR[Title/Abstract]) OR non coding RNA[Title/Abstract])) AND ((((((infection) OR infectious disease) OR virus) OR bacteria) OR fungus) OR parasite), limited to humans, up to 14^th^ July 2017. 56 out of 57 studies were included in these tables: one study was excluded because there was no RT-PCR confirmation of the microRNA profiling results. | | | | | | | | | |  |
| --- | --- | --- | --- | --- | --- | --- | --- | --- | --- | --- |
| **Infection** | Fluid | Cases | | Controls/ comparator | Candidate miRNA studied | Reference RNA in RT-PCR | Up-regulated miR(s) | Down-regulated miR(s) | Notes | Authors |
| **HBV** | serum | Occult HBV N=11  and chronic  HBV  N=30 | | Healthy controls  N=29 | 375, 92a,  10a, 223,  423, 23b/a,  342-3p, 99a,  122a, 125b,  150, let-7 | Spike in plant-miR156 | let-7c, 23b, 122, 150 |  | AUC of 4 miRNA signature combined = 1 | (Chen et al., 2012) |
| **HBV** | Serum | CHB, treatment  naive  N=115 | | Healthy controls  N=20 | 210 | Endogenous miR-16 | 210 |  | miR-210 correlated with necroinflamation | (Yu et al., 2015) |
| **HBV** | Serum | CHB, treatment naive  HBeAg+ve  N=79 | | Healthy controls HBeAg-ve  N=16 | 210 | Endogenous miR-16 | 210 |  |  |  |
| **HBV** | serum | Chronic HBV N=20 | | Healthy controls  N=20 | 122, 29 | U6 | 122, 29 |  |  | (Xing et al., 2014) |
| **HBV** | serum | Treatment naive CHB with  severe liver  cirrhosis  N=25 | | Treatment niave chronic HBV with  no/mild liver cirrhosis  N=36 | 29 | Cel-miR-3 | 29a, 29b,  29c |  |  | (Huang et al., 2014) |
| **HBV** | serum | Treatment naive  CHB  N=91 | | Healthy controls  N=12 | 29 | Cel-miR-3 | 29a, 29b,  29c |  |  |  |
| **HBV +HCV** | Serum | Dual infected HBV and HCV patients did not clear HBsAg after pegylated interferon and  ribavirin  N=30 | | Dual infected  HBV and HCV patients cleared HBsAg after pegylated  interferon and  ribavirin  N=91 | 122 | Endogenous 16 | 122 (at baseline pre treatment) |  |  | (Yen et al., 2016) |
| **HCV** | serum | Chronic HCV N=161 | | Healthy controls  N=40 | 146a-5p,  34a-5p,  130a-3p, 19a-3p, 192– 5p, 195–5p,  296–5p | SNORD68 | 34a, 130a,  19a, 192,  296, 195 | 146a | Candidate miRNA selected due to their association with interferon in the literature | (Motawi et al., 2015) |
| **HCV** | serum | HCV non  response to  PEG-  IFNa2b/RBV therapy  N=37 | | Chronic HCV  responder to  PEG-  IFNa2b/RBV therapy N=69 | 146a-5p,  34a-5p,  130a-3p, 19a-3p, 192– 5p, 195–5p,  296–5p | Endogenous  SNORD68 | 34a, 130a, 195 (pretreatment) | 192, 296 (pretreatment) | Candidate miRNA selected due to their association with interferon in the literature    Best AUC was 0.699 for 296, which was superior to the AUC for ALT, viral load, AFP |  |
| **HCV** | plasma | HCV N=50 | | Correlation study: RNA levels during acute infection | 122, 885, let-  7b, 494 | Spike in athmiR-159a |  | 122, let-7b Inversely correlated with HCV RNA  levels | Correlation between HCV RNA levels and miRNA levels in a longitudinal cohort study of HCV infection intravenous drug users | (El-Diwany et al., 2015) |
| **HCV** | serum | Hepatitis C  N=25 | | Healthy Control  N=25 | 122 | U6 | 122 |  | AUC = 0.929 for differentiating HCV and healthy controls | (Kumar et al., 2014) |
| **HCV** | serum | chronic HCV,  treatment naive  N=30 | | Healthy controls  N=12 | 155, 146a,  125b | RNU48 | 155, 125b,  146a, 122 |  | Increased levels of miR-155 also found in peripheral blood mononuclear cells | (Bala et al., 2012) |
| **HCV** | serum | | HCV N=60 | longitudinal response to  peg-IFN and ribovirin | 122 | Normalised to baseline line level 122 | 122 reduced during in treatment, then went back up in non-responders |  | Prospective cohort study  No correlation between miR-122 and HCV RNA at baseline. miR-122 did correlate with ALT and AST  miR-122 fell by week 4 of treatment, and fell more so in the responders versus the non-responders and increased after treatment in the relapsers and nonresponders compared to the sustained responders | (Köberle et al., 2013) |
| **HCV** | Serum | | Acute HCV (N=29) or  chronic HCV  without fibrosis  (N=18) | Healthy Controls  N=10 | based on RT-array  data from another analysis in the  92a, 20a | Spike in celmiR-39  Endogenous miR-574-3p (selected from array data) | 20a,  92a |  | Results are valid for both acute and chronic when analysed separately. Healthy controls versus acutely infected HCV patients: miR-20a AUC = 0.883±0.058 (95%CI=0.769–0.996) sensitivity 89.6%, specificity 80%, miR-92a AUC  0.889±0.057 (95%CI=0.778–1.001) sensitivity  89.6%,specificity 90%  Healthy controls versus chronic HCV infection: miR-20a AUC of 0.983±0.019 (95%CI=0.944–  1.022), sensitivity 100% , specificity of 80%, MiR92a AUC of 0.989±0.015 (95%CI=0.960–1.018) sensitivity 100% ,specificity 80% | (Shrivastava et al., 2013) |
| **HCV** | Serum | | Acute HCV that resolved  N=11 | Longitudinal changes during infection | 92a, 20a | Spike in celmiR-39 plus endogenous miR-574-3p (selected from array data) |  | 92a | No change seen in patients who progressed from acute to chronic N=18 No downregulation in miR20a seen as infection resolved, despite miR-20a being able to distinguish acute HCV infection compared to controls – could be a power issue. |  |
| **HCV** | serum | | HCV with advanced Fibrosis  N=58 | HCV with  mild fibrosis  N=106 | 122 | Spike in  SV40 |  | 122 | Correlation study miR-122 reduced in fibrosis but not supported by a direct comparison of mild versus severe fibrosis, therefore not a good biomarker of liver damage in HCV with respect to fibrosis | (Trebicka et al., 2013) |
| **HCV** | serum | | Chronic HCV N=102 | Healthy controls  N=24 | 122, 192 | ?Endogenous miR-191 not clear in the statistical analysis | 122, 192 |  |  | (van der Meer et al., 2013) |
| **HCV** | serum | | Chronic  Hepatitis B  N=89 | Healthy controls  N=19 | 122 | None | 122 |  |  | (Waidmann et al., 2012) |
| **HCV** | serum | | Chronic HCV Non responders to PEG IFN/ribavirin  N=24 | Chronic HCV Sustained response to  PEG  IFN/Ribavirin  N=28 | 122 | None |  |  | miR-122 not associated with response to treatment. Endogenous miR-16 purported to act as control, but not used to normalise miR122 results in a valid manner |  |
| **HCV** | Serum | | HCV did not respond to pegylated interferon and  ribavirin  N=28 | HCV  sustained response to pegylated  interferon and  ribavirin  N=98 | 122 | none |  | 122 |  | (Su et al., 2013) |
| **TB** | sputum + serum | | Pulmonary TB post treatment  levels  N=124 | Paired pre treatment levels | 144 | Endogenous U6 | 144 |  | Longitudinal study | (Lv et al., 2016) |
| **TB** | Serum | | Pulmonary TB N=75 | Healthy controls  N=52 | microarray | Endogenous U6 | 93*, 29a | 3125 | Pooled serum  miR-29a was also upregulated in saliva | (Fu et al., 2011) |
| **TB** | Serum | | Pulmonary TB N=75 | Healthy controls  N=52 | microarray | Endogenous U6 | 29a |  |  |  |
| **TB** | Serum | | Pulmonary TB N=50 | Healthy controls  N=85 | RT-PCR  arrays | Endogenous miR-16 | 361-5p,  889, 567-  3p, 210,  26a, 432,  134, |  | AUC = 0.863 differentiating pulmonary TB from healthy controls | (Qi et al., 2012) |
| **TB** | Serum | | Pulmonary TB N=50 | Enterovirus, pertussis, varicella  N=60 | RT-PCR  arrays | Endogenous miR-16 | 361-5p,  889, 567-  3p, 210,  26a, 432, 134 |  | miR-26a, miR-432, miR-134 signature gave AUC =0.7 for differentiating pulmonary TB from the enterovirus/pertussis/ varicella group |  |
| **TB** | Sputum | | Pulmonary Tb N= 58 | Healthy controls  N=32 | Microarrays | U6 | 3197, 147 | 19b-2* |  | (Yi et al., 2012) |
| **TB** | Serum | | TB treatment naive  N=30 | Healthy controls  N=3- | 16, 29a, 125b, 155 | None | 16 | 155 | The AUC for miR-16 = 1, miR-0.967 for diagnosing TB.  miR-16 and miR-155 levels normalised once on treatment | (Wagh et al., 2017) |
| **HIV** | Serum | | HIV on ART  non survivors N=126 | HIV on ART survivors  N=247 | 126, let7e,  21, 24, 122,  134,145,200a  , 150,  221,223, 31,  370, 29a,  146a, 197,  155 | Endogenous miR-16 |  |  | No association with mortality | (Murray et al., 2015) |
| **Sepsis** | serum | | septic patients on ITU N=137 | non septic  patients on  ITU  N=84 | 223 | Spike in SV50 |  |  | miR-223 did not identify sepsis or predict survival in ITU | (Benz et al., 2015) |
| **Sepsis** | serum | | Sepsis N=138 | other critically  ill patients  N=85 | 150 | spiked in SV41 |  |  | no effect for miR-150  contradicted findings from their much smaller pilot study | (Roderburg et al., 2013) |
| **sepsis in** | serum | | Sepsis N=138 | Critically ill  non septic  N=85 | 133a | Spike in  miRNA mimic SV40 | 133a |  | Linked to Roderburg study  AUC for 133a = 0.609 in identification of sepsis in critically ill patients which is substantially inferior to AUC for CRP or procalcitonin | (Tacke et al., 2014) |
| **Sepsis** **ICU** | Serum | | Septic shock or  severe sepsis  N=123 | Mild sepsis N=43 | 223, 15b,  483-5p,  193b, 122,  499-5p | U6 |  | 15b, 223,  483-5p,  499 | miR-499-5p was the best at distinguishing mild sepsis from septic shock AUC | (Wang et al., 2012b) |
| **Sepsis** | Serum | | Mild Sepsis N=43 | Healthy controls  N=24 | 223, 15b,  483-5p,  193b, 122,  499-5p | U6 | 223, 15b,  483-5p | 193b |  |  |
| **sepsis** | serum | | Septic in ICU N=50 | Systemic Inflammatory syndrome in  ICU  N=30 | 132, 146a,  155,  223,15b ,  126, let-7 | Spike in  mmu-miR295 |  | 146a, 223 |  | (Wang et al., 2010) |
| **sepsis** | Serum | | Septic in ICU N=50 | Healthy controls  N=20 | 132, 146a,  155, 223, 15b , 126, let-7 | Spike in  mmu-miR295 |  | 146a, 223,  126 | Candidates identified from in-vitro miRNA response in dendritic cells stimulated with LPS |  |
| **sepsis** | serum | | Sepsis survivors  N=12 | Sepsis non-survivors  N=12 | Microarray | Endogenous 5sRNA | 297 | 574-5p | Best AUC for a single microRNA was miR-5745p: AUC 0.736 | (Wang et al., 2012a) |
| **Sepsis**  **Sepsis** | Serum  Serum | | Sepsis  N=103 | Systemic inflammatory  response N=95 | 143 | Endogenous U6 |  |  |  | (Han et al., 2016) |
|  |  |  | Sepsis N=103 | Healthy controls  N=40 | 143 | Endogenous U6 | 143 |  | AUC for miR-143 (AUC=0.91, CI 0.86, 0.95) was higher than for CRP and procalcitonin |  |
| **Sepsis** | Plasma | | Severe sepsis  with shock  N=62 | Sepsis without shock N=32 | 15a,16,34a,  126,27a,150  223, 181b,  155, 125b,  146a, 486, 21 | Endogenous U6 | 34a, 15a | 27a | AUC for predicting shock when all miRNA combined = 0.78 (CI 0.66-0.9) | (Goodwin et al., 2015) |
| **Hand foot and mouth** | serum | | EV71 N=46 | Coxsackie N=24 | 148a, 6283p, 143, 324-  3p, 206, 1405p, 455-5p  362-3p | Cel-miR-238 |  | 148a, 143,  324-3p,  545, 140-  5p | The AUC when miR-545, miR-324-3p, miR-143 = 0.761 (95% CI = 0.644–0.879) Optimal sensitivity 0.792 and specificity 0.652.  Candidate miRNA chosen based on RT-PCR arrays from EV71 versus healthy control analysis | (Cui et al., 2011) |
| **flu vaccine** | serum and serum exosomes | | Post flu vaccine  (paired samples)  N=106 | pre-flu vaccine (paired samples) | 150 | Endogenous U6 | 150 |  | miR-150 upregulated after single dose in adults and  2^nd^ dose in children - ? relates to a memory response. miR-150 upregulation corresponded to antibody titres in adults. Upregulation of miR-150 more pronounced in exosomes  Best AUC for predicting vaccination status was in the exosomal analysis: AUC= 0.75. | (de Candia et al., 2013) |
| **Shistosomiasis** | serum | | Schistosomiasis N = 9 | Healthy controls  N=5 | 146b, 122,  223, 199a5p, 199a-3p,  34a | Spike in  Cel-miR-39 | 223 |  |  | (He et al., 2013) |
| **Malaria** | plasma | | Malaria – plasmodium vivax  N=16 | Healthy controls  N=19 | 451, 16, 223,  191*, 296-  3p, let-7a,  409-3p | Spike in  Cel-miR-39 | 451, 16 |  |  | (Chamnanchanunt et al., 2015) |

**References for Supplementary Material**

Bala, S., Tilahun, Y., Taha, O., Alao, H., Kodys, K., Catalano, D., et al. (2012). Increased microRNA-155 expression in the serum and peripheral monocytes in chronic HCV infection. *J. Transl. Med.* 10, 151. doi:10.1186/1479-5876-10-151.

Benz, F., Tacke, F., Luedde, M., Trautwein, C., Luedde, T., Koch, A., et al. (2015). Circulating microRNA-223 serum levels do not predict sepsis or survival in patients with critical illness. *Dis. Markers* 2015, 384208. doi:10.1155/2015/384208.

Chamnanchanunt, S., Kuroki, C., Desakorn, V., Enomoto, M., Thanachartwet, V., Sahassananda, D., et al. (2015). Downregulation of plasma miR-451 and miR-16 in Plasmodium vivax infection. *Exp. Parasitol.* 155, 19–25. doi:10.1016/j.exppara.2015.04.013.

Chen, Y., Li, L., Zhou, Z., Wang, N., Zhang, C.-Y., and Zen, K. (2012). A pilot study of serum microRNA signatures as a novel biomarker for occult hepatitis B virus infection. *Med. Microbiol. Immunol.* 201, 389–95. doi:10.1007/s00430-011-0223-0.

Cui, L., Qi, Y., Li, H., Ge, Y., Zhao, K., Qi, X., et al. (2011). Serum microRNA expression profile distinguishes enterovirus 71 and coxsackievirus 16 infections in patients with hand-foot-and-mouth disease. *PLoS One* 6, e27071. doi:10.1371/journal.pone.0027071.

de Candia, P., Torri, A., Gorletta, T., Fedeli, M., Bulgheroni, E., Cheroni, C., et al. (2013). Intracellular modulation, extracellular disposal and serum increase of MiR-150 mark lymphocyte activation. *PLoS One* 8, e75348. doi:10.1371/journal.pone.0075348.

El-Diwany, R., Wasilewski, L. N., Witwer, K. W., Bailey, J. R., Page, K., Ray, S. C., et al. (2015). Acute Hepatitis C Virus Infection Induces Consistent Changes in Circulating MicroRNAs That Are Associated with Nonlytic Hepatocyte Release. *J. Virol.* 89, 9454–64. doi:10.1128/JVI.00955-15.

Fu, Y., Yi, Z., Wu, X., Li, J., and Xu, F. (2011). Circulating microRNAs in patients with active pulmonary tuberculosis. *J. Clin. Microbiol.* 49, 4246–51. doi:10.1128/JCM.05459-11.

Goodwin, A. J., Guo, C., Cook, J. A., Wolf, B., Halushka, P. V, and Fan, H. (2015). Plasma levels of microRNA are altered with the development of shock in human sepsis: an observational study. *Crit. Care* 19, 440. doi:10.1186/s13054-015-1162-8.

Han, Y., Dai, Q.-C., Shen, H.-L., and Zhang, X.-W. (2016). Diagnostic value of elevated serum miRNA-143 levels in sepsis. *J. Int. Med. Res.* 44, 875–81. doi:10.1177/0300060516645003.

He, X., Sai, X., Chen, C., Zhang, Y., Xu, X., Zhang, D., et al. (2013). Host serum miR-223 is a potential new biomarker for Schistosoma japonicum infection and the response to chemotherapy. *Parasit. Vectors* 6, 272. doi:10.1186/1756-3305-6-272.

Huang, C., Zheng, J. M., Cheng, Q., Yu, K. K., Ling, Q. X., Chen, M. Q., et al. (2014). Serum microRNA-29 levels correlate with disease progression in patients with chronic hepatitis B virus infection. *J. Dig. Dis.* 15, 614–21. doi:10.1111/1751-2980.12185.

Köberle, V., Waidmann, O., Kronenberger, B., Andrei, A., Susser, S., Füller, C., et al. (2013). Serum microRNA-122 kinetics in patients with chronic hepatitis C virus infection during antiviral therapy. *J. Viral Hepat.* 20, 530–5. doi:10.1111/jvh.12075.

Kumar, S., Chawla, Y. K., Ghosh, S., and Chakraborti, A. (2014). Severity of hepatitis C virus (genotype-3) infection positively correlates with circulating microRNA-122 in patients sera. *Dis. Markers* 2014, 435476. doi:10.1155/2014/435476.

Lv, Y., Guo, S., Li, X.-G., Chi, J.-Y., Qu, Y.-Q., and Zhong, H.-L. (2016). Sputum and serum microRNA-144 levels in patients with tuberculosis before and after treatment. *Int. J. Infect. Dis.* 43, 68–73. doi:10.1016/j.ijid.2015.12.014.

Motawi, T. K., Shaker, O. G., El-Maraghy, S. A., and Senousy, M. A. (2015). Serum interferon-related microRNAs as biomarkers to predict the response to interferon therapy in chronic hepatitis C genotype 4. *PLoS One* 10, e0120794. doi:10.1371/journal.pone.0120794.

Murray, D. D., Suzuki, K., Law, M., Trebicka, J., Neuhaus, J., Wentworth, D., et al. (2015). Circulating microRNAs in Sera Correlate with Soluble Biomarkers of Immune Activation but Do Not Predict Mortality in ART Treated Individuals with HIV-1 Infection: A Case Control Study. *PLoS One* 10, e0139981. doi:10.1371/journal.pone.0139981.

Qi, Y., Cui, L. L. L. L., Ge, Y., Shi, Z., Zhao, K., Guo, X., et al. (2012). Altered serum microRNAs as biomarkers for the early diagnosis of pulmonary tuberculosis infection. *BMC Infect. Dis.* 12, 384. doi:10.1186/1471-2334-12-384.

Roderburg, C., Luedde, M., Vargas Cardenas, D., Vucur, M., Scholten, D., Frey, N., et al. (2013). Circulating microRNA-150 serum levels predict survival in patients with critical illness and sepsis. *PLoS One* 8, e54612. doi:10.1371/journal.pone.0054612.

Shrivastava, S., Petrone, J., Steele, R., Lauer, G. M., Di Bisceglie, A. M., and Ray, R. B. (2013). Up-regulation of circulating miR-20a is correlated with hepatitis C virus-mediated liver disease progression. *Hepatology* 58, 863–71. doi:10.1002/hep.26296.

Su, T.-H., Liu, C.-H., Liu, C.-J., Chen, C.-L., Ting, T.-T., Tseng, T.-C., et al. (2013). Serum microRNA-122 level correlates with virologic responses to pegylated interferon therapy in chronic hepatitis C. *Proc. Natl. Acad. Sci.* 110, 7844–7849. doi:10.1073/pnas.1306138110.

Tacke, F., Roderburg, C., Benz, F., Cardenas, D. V., Luedde, M., Hippe, H.-J., et al. (2014). Levels of circulating miR-133a are elevated in sepsis and predict mortality in critically ill patients. *Crit. Care Med.* 42, 1096–104. doi:10.1097/CCM.0000000000000131.

Trebicka, J., Anadol, E., Elfimova, N., Strack, I., Roggendorf, M., Viazov, S., et al. (2013). Hepatic and serum levels of miR-122 after chronic HCV-induced fibrosis. *J. Hepatol.* 58, 234–9. doi:10.1016/j.jhep.2012.10.015.

van der Meer, A. J., Farid, W. R. R., Sonneveld, M. J., de Ruiter, P. E., Boonstra, A., van Vuuren, A. J., et al. (2013). Sensitive detection of hepatocellular injury in chronic hepatitis C patients with circulating hepatocyte-derived microRNA-122. *J. Viral Hepat.* 20, 158–66. doi:10.1111/jvh.12001.

Wagh, V., Urhekar, A., and Modi, D. (2017). Levels of microRNA miR-16 and miR-155 are altered in serum of patients with tuberculosis and associate with responses to therapy. *Tuberculosis (Edinb).* 102, 24–30. doi:10.1016/j.tube.2016.10.007.

Waidmann, O., Bihrer, V., Pleli, T., Farnik, H., Berger, A., Zeuzem, S., et al. (2012). Serum microRNA-122 levels in different groups of patients with chronic hepatitis B virus infection. *J. Viral Hepat.* 19, e58-65. doi:10.1111/j.1365-2893.2011.01536.x.

Wang, H., Meng, K., Chen, W. jun, Feng, D., Jia, Y., and Xie, L. (2012a). Serum miR-574-5p: a prognostic predictor of sepsis patients. *Shock* 37, 263–7. doi:10.1097/SHK.0b013e318241baf8.

Wang, H., Zhang, P., Chen, W., Feng, D., Jia, Y., and Xie, L. (2012b). Four serum microRNAs identified as diagnostic biomarkers of sepsis. *J. Trauma Acute Care Surg.* 73, 850–4. doi:10.1097/TA.0b013e31825a7560.

Wang, J., Yu, M., Yu, G., Bian, J., Deng, X., Wan, X., et al. (2010). Serum miR-146a and miR-223 as potential new biomarkers for sepsis. *Biochem. Biophys. Res. Commun.* 394, 184–8. doi:10.1016/j.bbrc.2010.02.145.

Xing, T. J., Jiang, D. F., Huang, J. X., and Xu, Z. L. (2014). Expression and clinical significance of miR-122 and miR-29 in hepatitis B virus-related liver disease. *Genet. Mol. Res.* 13, 7912–8. doi:10.4238/2014.September.29.4.

Yen, Y.-H., Huang, C.-M., Wei, K.-L., Wang, J.-H., Lu, S.-N., Lee, C.-M., et al. (2016). MicroRNA-122 as a predictor of HBsAg seroclearance in hepatitis B and C dual infected patients treated with interferon and ribavirin. *Sci. Rep.* 6, 33816. doi:10.1038/srep33816.

Yi, Z., Fu, Y., Ji, R., Li, R., and Guan, Z. (2012). Altered microRNA Signatures in Sputum of Patients with Active Pulmonary Tuberculosis. *PLoS One* 7, e43184. doi:10.1371/journal.pone.0043184.

Yu, F., Yang, J., Ouyang, J., Zheng, Y., Chen, B., Li, G., et al. (2015). Serum microRNA-210 levels in different groups of chronic hepatitis B patients. *Clin. Chim. Acta.* 450, 203–9. doi:10.1016/j.cca.2015.08.022.

**
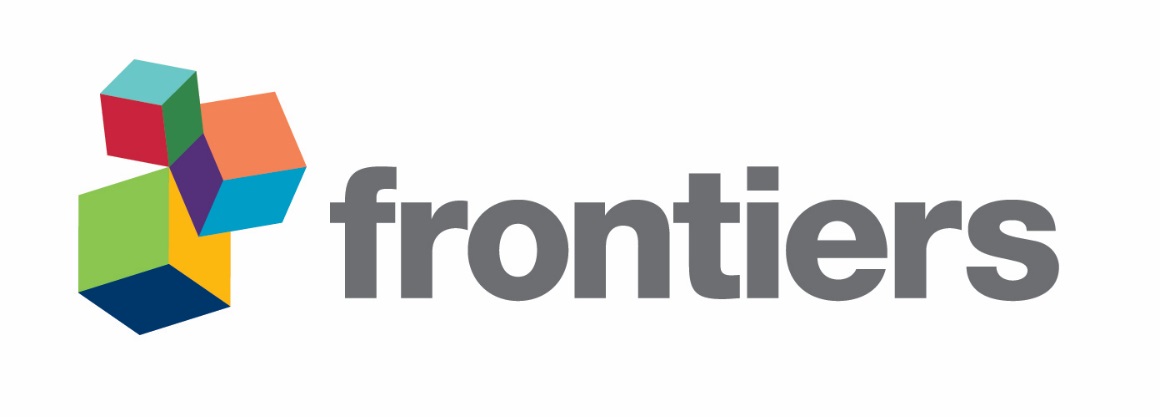
**
